# Supplementary material for: Alteration of pro-carcinogenic gut microbiota is associated with clear cell renal cell carcinoma tumorigenesis
Source: Front Microbiol. 2023 Apr 5;14:1133782. doi: 10.3389/fmicb.2023.1133782 (PMC10113506; doi:10.3389/fmicb.2023.1133782)
Supplement: Supplementary file 1 [file Data_Sheet_1.docx]

**SUPPLEMENTAL MATERIAL**

**
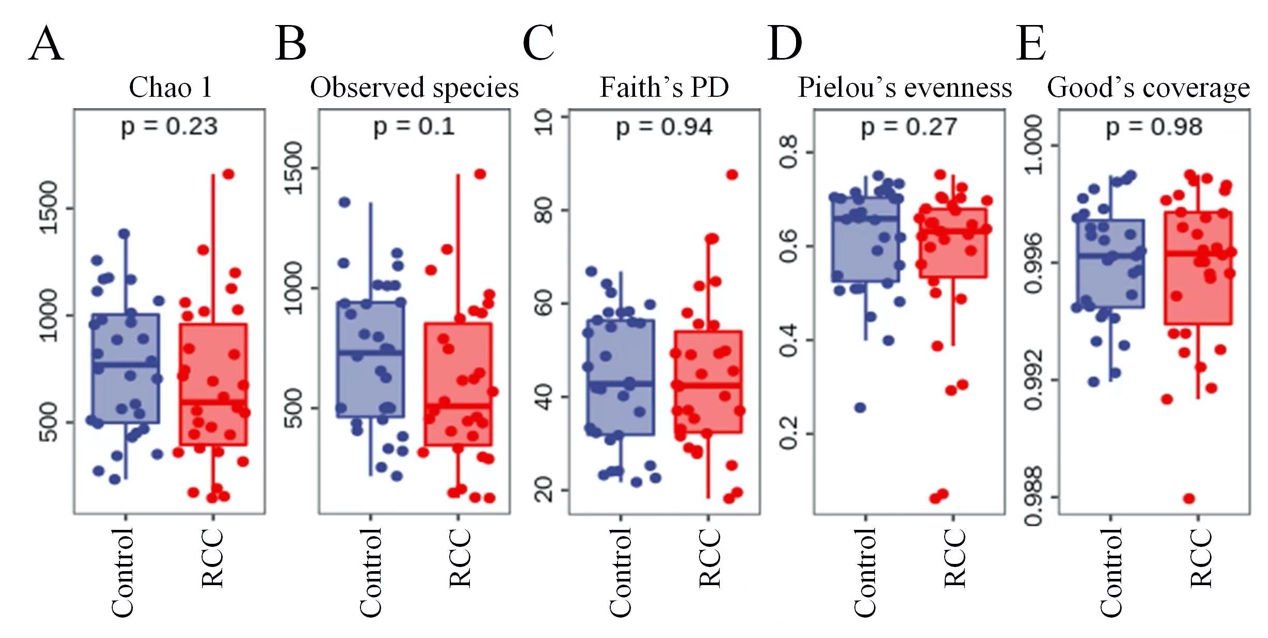
**

**Figure S1 Alpha diversity indices in the RCC group and the Control group were measured based on Chao 1 (A), Observed species (B), Faith’s PD (C), Pielou’s evenness (D), and Good’s coverage (E).** Blue represents Control group, red RCC group. Non-significant P>0.05.

**
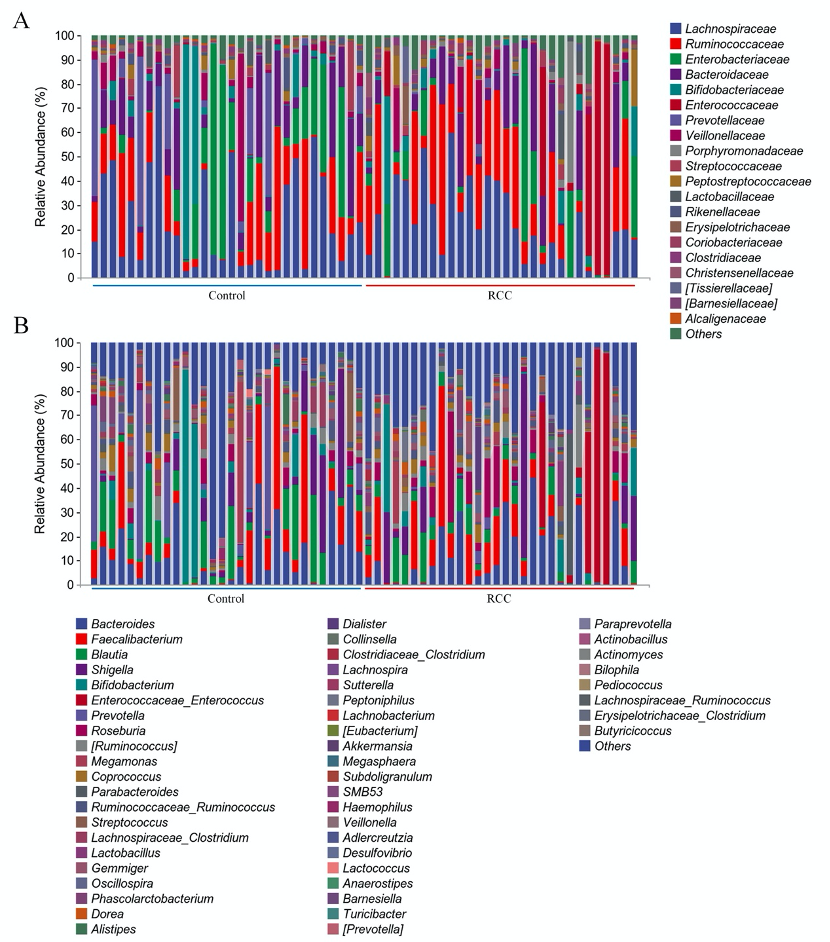
**

**Figure S2 Relative abundance of gut microbiota in the RCC group and the Control group at family (A) and phyla (B) level taxonomically.**

**
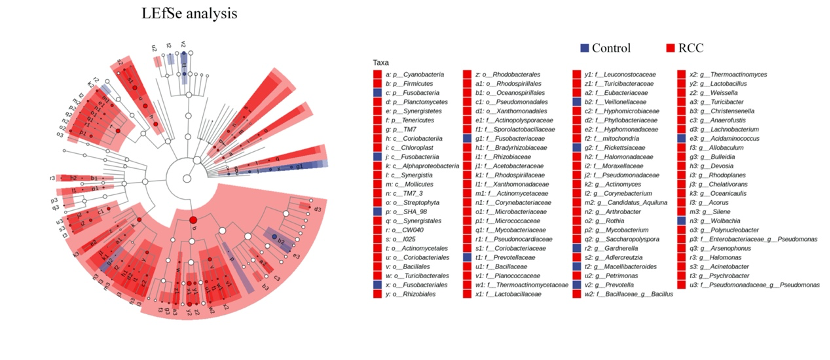
**

**Figure S3 LEfSe was used to analyze the abundance of ASVs in the two groups.**

**
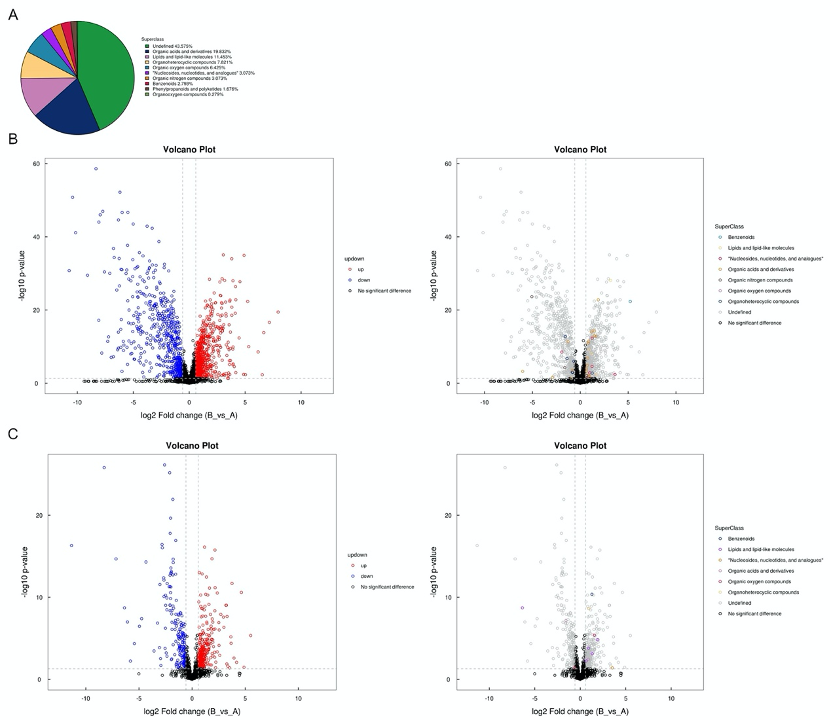
**

**Figure S4 Differential blood metabolites were identified and classified .** (A) The sector graph showed the proportion of serum metabolites based on superclass. Alteration profiles of metabolites in the two groups analyzed by volcano plots in positive ion mode (B) and negative ion mode (C) respectively.

**
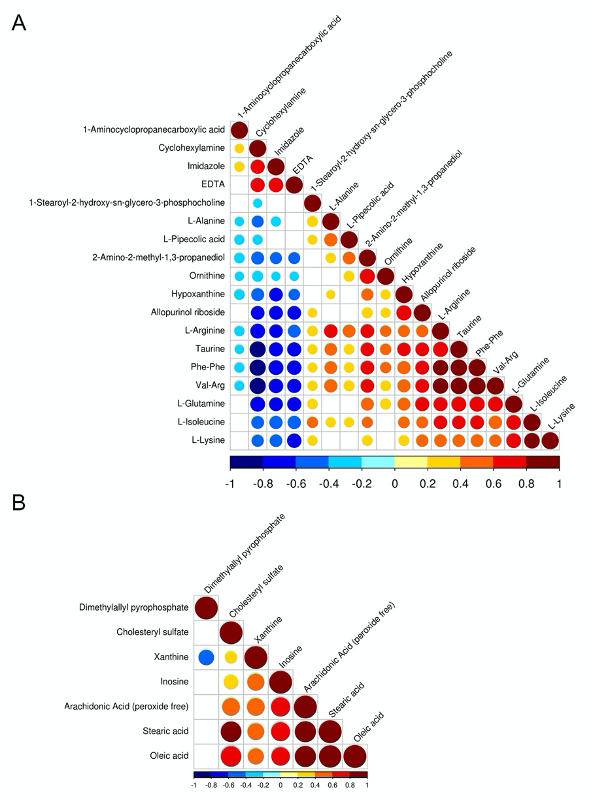
**

**Figure S5 Spearman analysis illustrated the correlation between altered metabolites in positive (A) and negative (B) ion mode.**

**
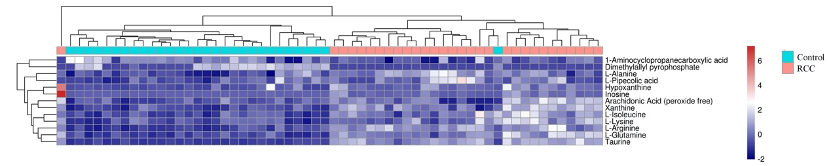
**

**Figure S6** **Changes in abundance of genes corresponding to the metabolic pathway in the two groups.**

**
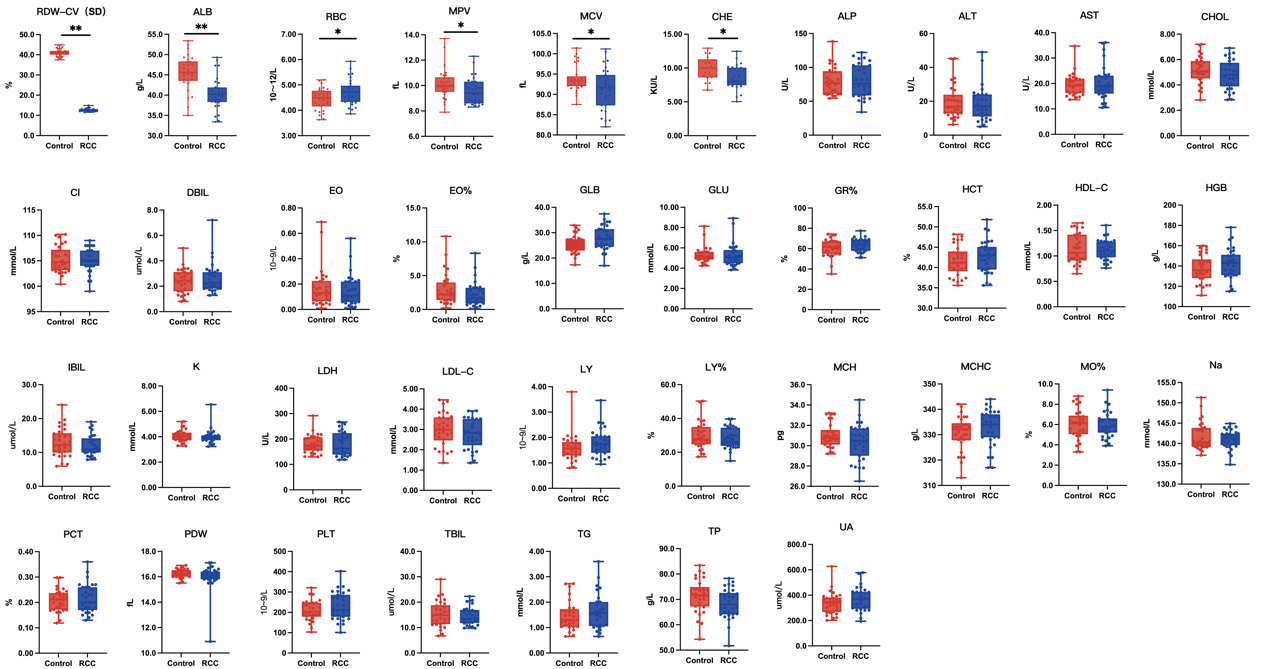
**

**Figure S7 Comparisons of clinical indices based on blood routine tests, liver and kidney function and electrolyte detections.** **Abbreviations:** RDW-CV (SD), red cell volume distribution width; ALB, albumin; RBC, red blood cell; MPV, mean platelet volume; MCV, mean corpuscular volume; CHE, Cholinesterase; ALP, alkaline phosphatase; ALT, alanine transaminase; AST, aspartate transaminase; CHOL, cholesterol; CI, chlorine; DBIL, direct bilirubin; EO, eosinophil; EO%, eosinophil%; GLB, globulin; GLU, glucose; GR%, granulocyte%; HCT, red blood cell specific volume; HDL-C, High-density lipoprotein cholesterol; HGB, hemoglobin; IBIL, indirect bilirubin; K, kalium; LDH, lactate dehydrogenase; LDL-C, low-density lipoprotein cholesterol; LY, lymphocyte; LY%, lymphocyte%; MCH, mean corpuscular hemoglobin; MCHC, mean corpuscular hemoglobin concentration; MO%, monocyte%; Na, natrium; PCT, procalcitonin; PDW, platelet distribution width; PLT, platelet; TBIL, total bilirubin; TG, triglyceride; TP, total protein; UA, uric acid.

**Table S1 22 inflammatory transcripts were listed based on overlap between univariate Cox regression and different expression analysis.**

**Table S2 202 metabolism-related transcripts were listed based on overlap between univariate Cox regression and different expression analysis.**
