# Supplementary material for: Alteration of pro-carcinogenic gut microbiota is associated with clear cell renal cell carcinoma tumorigenesis
Source: Front Microbiol. 2023 Apr 5;14:1133782. doi: 10.3389/fmicb.2023.1133782 (PMC10113506; doi:10.3389/fmicb.2023.1133782)
Supplement: Supplementary file 2 [file Table_1.pdf]

99 inflammatory genes with significant prognosis were listed by univariate Cox regression model

id	HR	HR.95L	HR.95H	pvalue
FZD5	0.9129107	0.86883708	0.95922006	0.00030728
NMI	1.05470797	1.00320666	1.10885319	0.03704159
STAB1	1.01875199	1.00376194	1.0339659	0.01403226
CCL5	1.00320496	1.00127234	1.00514132	0.0011445
CXCL8	1.00907717	1.00438722	1.01378902	0.0001437
SLC31A1	0.94832823	0.90739702	0.99110579	0.01843129
BTG2	0.99137504	0.98485091	0.9979424	0.0101293
CCL22	0.70513182	0.52572009	0.94577111	0.01969325
BEST1	1.45837845	1.22380524	1.73791356	2.47E-05
EREG	1.12632441	1.07067752	1.18486346	4.19E-06
ABCA1	0.96967483	0.94308401	0.9970154	0.029957
IRF7	1.04610027	1.02986261	1.06259395	1.64E-08
NAMPT	1.02269872	1.01113337	1.03439636	0.00010973
LAMP3	1.1326341	1.0117205	1.26799844	0.03059798
PLAUR	1.06435766	1.04827885	1.08068309	9.67E-16
BST2	1.00154156	1.00040227	1.00268215	0.00798888
P2RY2	1.63208984	1.25908914	2.11559068	0.0002154
MXD1	1.07979072	1.01031924	1.1540392	0.02366395
IL2RB	1.03013997	1.00247208	1.0585715	0.03254044
TAPBP	1.00487343	1.00084788	1.00891518	0.01760667
IL15RA	1.10538281	1.06528042	1.14699485	1.07E-07
TNFRSF9	1.06175986	1.0032676	1.12366232	0.03819084
CDKN1A	0.99495113	0.99078321	0.99913657	0.01811453
ICAM1	1.01181784	1.00459476	1.01909285	0.0013086
PSEN1	0.92491372	0.86115094	0.99339773	0.03221584
ADORA2B	1.35840351	1.23134863	1.49856836	9.74E-10
CXCR6	1.1036578	1.0192389	1.19506873	0.01512666
GNAI3	0.84751286	0.72283863	0.99369073	0.04155746
ACVR2A	0.59293567	0.48873807	0.71934792	1.15E-07

47 different expression genes associated with inflammation were listed.

gene	LogFC	fdr
ABCA1	-2.953	1.57E-49
AXL	-1.875	1.43E-37
BEST1	-1.187	4.34E-21
C3AR1	-1.533	2.04E-11
C5AR1	-1.437	5.08E-17
CCL2	-3.263	5.74E-36
CD14	-2.528	2.97E-15
CD55	-1.437	2.85E-15
CD82	1.024	1.67E-06
CLEC5A	-1.109	4.81E-17
CMKLR1	-1.718	5.08E-32
CSF1	-2.547	1.87E-31
CSF3R	-2.012	4.11E-31
CYBB	-2.069	1.12E-18
F3	-1.36	1.30E-13
FPR1	-1.925	2.47E-23
FZD5	-1.045	2.27E-21
GABBR1	-2.219	7.01E-47
GPC3	-1.451	3.36E-14
ICAM1	-1.277	1.01E-09
IL10	-1.009	1.59E-19
IL10RA	-1.888	1.01E-27
IL18	-1.244	6.94E-07
IL18R1	-1.122	5.34E-19
IL1R1	-1.294	9.80E-14
IL4R	-2.62	9.13E-44
IRF7	-1.056	4.84E-10
LCP2	-1.416	6.90E-22
LPAR1	-1.825	5.07E-19

Overlapping

FZD5
STAB1
BEST1
ABCA1
IRF7
ICAM1
IL4R
IL10RA
CLEC5A
MARCO
TLR2
CSF3R
CD14
AXL
F3
OSMR
GABBR1
PDPN
FPR1
CSF1
CD82
PTGIR

CSF3	1.23891784	1.13111368	1.35699658	3.98E-06
IL4R	1.02934518	1.00428093	1.05503496	0.02147051
ITGA5	1.00943727	1.0015674	1.01736897	0.01866478
IL10RA	1.0263015	1.00376331	1.04934575	0.02193388
ROS1	1.18984359	1.02291524	1.38401278	0.02421359
IFITM1	1.00600409	1.00362704	1.00838677	7.07E-07
DCBLD2	1.02339603	1.01581354	1.03103512	1.09E-09
HRH1	1.07954281	1.01504804	1.14813549	0.01488452
IL1B	1.06351629	1.00375128	1.12683979	0.0369017
CLEC5A	1.11065422	1.02392804	1.20472606	0.01140618
TNFSF15	0.71083468	0.51786944	0.9757014	0.03467016
MARCO	1.0277726	1.01117864	1.04463887	0.00097194
TLR2	1.04784002	1.02147518	1.07488535	0.00032541
RIPK2	1.05957533	1.03463649	1.0851153	1.92E-06
PVR	1.02465486	1.0061689	1.04348046	0.00874053
CSF3R	1.04570184	1.01792836	1.07423309	0.00113884
RGS1	1.00590403	1.0013769	1.01045162	0.0105326
CD14	1.00482226	1.00224112	1.00741006	0.00024654
EDN1	0.9900498	0.9831669	0.99698088	0.00496262
ATP2C1	0.96090567	0.92791772	0.99506635	0.0252565
KIF1B	0.78526379	0.71176784	0.8663488	1.43E-06
NFKB1	0.91887109	0.88200439	0.95727878	5.13E-05
IRF1	1.0192369	1.00130752	1.03748733	0.03535517
KIAA1429	0.86585751	0.79019972	0.94875917	0.00201857
AXL	1.01553293	1.00659363	1.02455162	0.00063357
LTA	1.38253074	1.1152997	1.7137916	0.00311957
F3	1.01271114	1.00634904	1.01911345	8.55E-05
LIF	1.01240207	1.00595261	1.01889287	0.00015675
OSMR	1.01174039	1.00162349	1.02195948	0.02282636
RHOG	1.02619384	1.01351018	1.03903623	4.61E-05
IFNGR2	1.01753546	1.00662135	1.0285679	0.00158086
SGMS2	0.88679685	0.84107703	0.93500194	8.65E-06

LY6E	1.594	1.11E-14
LYN	-1.091	3.13E-11
MARCO	-3.537	6.62E-29
MSR1	-1.523	9.54E-19
MYC	-1.759	7.61E-21
OSMR	-1.402	3.17E-20
PDPN	-1.389	4.58E-08
PIK3R5	-1.85	2.10E-42
PTGER4	-1.714	4.02E-37
PTGIR	-1.774	1.25E-35
PTPRE	-1.238	1.02E-14
SCN1B	-1.805	7.75E-19
SEMA4D	-1.295	3.05E-26
SLC7A2	-1.539	6.08E-15
STAB1	-2.657	9.19E-33
TLR2	-1.14	1.38E-18
TNFSF10	-1.674	3.43E-07
TNFSF9	1.145	1.40E-28

AQP9	1.01727713	1.00658399	1.02808387	0.00148736
GABBR1	1.20414533	1.13830749	1.27379113	9.45E-11
ABI1	0.95180069	0.91334454	0.99187602	0.01889398
SLC4A4	0.97445195	0.96215787	0.98690313	6.47E-05
TPBG	1.0855089	1.01414974	1.16188915	0.01803234
HAS2	1.23402023	1.1383703	1.33770701	3.25E-07
TACR1	0.50682944	0.29138024	0.88158373	0.01611842
NOD2	1.44320575	1.26123428	1.65143214	9.55E-08
CXCL10	1.00312214	1.00035385	1.00589809	0.02704419
SPHK1	1.08272245	1.050611	1.11581538	2.29E-07
CX3CL1	0.97850683	0.97047577	0.98660436	2.38E-07
GPR132	1.19395188	1.06283779	1.34124051	0.00281937
SLAMF1	1.31152696	1.07759291	1.59624562	0.00682038
PDPN	1.03651981	1.02291083	1.05030984	1.04E-07
CALCRL	0.96766322	0.95386565	0.98166036	7.25E-06
FPR1	1.0302474	1.00931101	1.05161808	0.00444515
CSF1	1.04565562	1.02751396	1.06411758	5.75E-07
ADRM1	1.0307723	1.01994768	1.0417118	1.83E-08
SERPINE1	1.00094695	1.00053767	1.00135641	5.74E-06
PTGER2	1.05139359	1.02014303	1.08360147	0.00113244
EBI3	1.06767353	1.03859852	1.09756248	3.34E-06
GNA15	1.15313249	1.09896839	1.20996614	6.45E-09
EMP3	1.01287184	1.00733809	1.01843599	4.75E-06
MMP14	1.00465099	1.00267287	1.006633	3.94E-06
AHR	0.97729905	0.95701508	0.99801295	0.03188598
ADGRE1	1.14061015	1.05000307	1.23903591	0.00183718
APLNR	0.98064422	0.96962805	0.99178555	0.00069642
CD82	1.0361847	1.0224981	1.0500545	1.61E-07
SLC31A2	0.64934855	0.5113584	0.82457537	0.00039645
IFNAR1	0.94643823	0.92199545	0.97152902	3.73E-05
TIMP1	1.00085027	1.00058102	1.0011196	5.97E-10
CHST2	1.03042977	1.00466138	1.05685908	0.02034761

TNFRSF1B	1.01468474	1.00113834	1.02841444	0.03351402
TLR3	0.96208377	0.94392336	0.98059356	7.02E-05
PTGIR	1.10382726	1.00996216	1.20641611	0.02936261
CCL7	1.33885097	1.21415716	1.47635081	4.91E-09
OSM	1.09411646	1.04236656	1.14843556	0.00027434
KLF6	0.99146337	0.98726197	0.99568264	7.59E-05
