# Supplementary material for: Alteration of pro-carcinogenic gut microbiota is associated with clear cell renal cell carcinoma tumorigenesis
Source: Front Microbiol. 2023 Apr 5;14:1133782. doi: 10.3389/fmicb.2023.1133782 (PMC10113506; doi:10.3389/fmicb.2023.1133782)
Supplement: Supplementary file 3 [file Table_2.pdf]

800 metabolic genes with significant prognosis were listed by univariate Cox regression model

484 different expression genes associated with metabolism were listed.

Overlapping

id	HR	HR.95L	HR.95H	pvalue	gene	treatMean	conMean	logFC	pValue	fdr	HS6ST2
ACOX1	0.868928758	0.814146372	0.927397348	2.35E-05	ACADSB	8.133489794	31.33014843	-1.945605205	2.12E-35	3.75E-34	DBT
CSNK1G2	1.068343957	1.02648998	1.111904483	0.001186143	SLC5A8	9.818593799	3.18239975	1.625401351	0.005550213	0.006473892	PRKAR2B
BST1	0.882717252	0.807118924	0.965396454	0.006316588	PSMA8	0.021323129	0.00790015	1.43246718	8.41E-08	1.26E-07	HADH
PSMC4	1.032892638	1.016579098	1.049467969	6.77E-05	ABCB11	0.023718616	0.002771241	3.097415619	0.000492256	0.000606646	CROT
ETFDH	0.882204756	0.837014195	0.929835165	2.99E-06	ELOVL3	0.137365008	0.38724607	-1.495236051	2.12E-24	7.85E-24	EPM2A
HSD17B10	1.007385677	1.000208918	1.014613931	0.043671561	FABP6	19.45977802	0.390912925	5.637504156	1.50E-38	9.55E-37	PIK3R6
LHPP	1.058747097	1.018425929	1.100664648	0.003956555	UGT8	7.249328525	24.84969878	-1.777309088	9.39E-32	7.66E-31	PTPN13
ACADSB	0.882560074	0.844383262	0.92246296	3.07E-08	SYT5	0.072761991	0.013979423	2.379880161	1.93E-09	3.13E-09	FDX1
SLC5A8	0.979318255	0.964484892	0.994379748	0.00728047	B3GAT2	0.130843417	0.058100202	1.171226254	3.79E-12	6.94E-12	PSMB9
MCCC2	0.945646309	0.914703359	0.977636009	0.000993189	ABCD1	6.661305298	3.263849139	1.02923053	1.62E-31	1.25E-30	NDST3
RPL38	1.003057625	1.000565837	1.005555617	0.016140881	PLA2G4C	3.575800503	1.71507365	1.059995719	1.61E-20	4.59E-20	FDFT1
SCLY	1.862242349	1.005647752	3.448470462	0.047942373	SLC2A5	18.04480087	5.262929739	1.777645183	4.95E-17	1.12E-16	TYMP
ELOVL3	1.942013973	1.150968325	3.276735067	0.012891271	PRELP	8.212714025	16.86733319	-1.038300927	1.21E-13	2.34E-13	GAPDH
TNFRSF21	0.988740179	0.981745431	0.995784763	0.001771297	FDXR	7.781235188	2.955498444	1.396597725	5.67E-32	4.88E-31	ENO2
UGT8	0.96393211	0.9332732	0.995598193	0.02591617	PHGDH	6.076919757	15.55599317	-1.356058359	6.45E-26	2.76E-25	SUCLG2
HS3ST3A1	1.089433001	1.053884105	1.126181009	4.18E-07	ATP5S	2.821232472	5.713454486	-1.018037746	4.10E-30	2.66E-29	TYMS
CYP7B1	0.894624551	0.835288727	0.958175372	0.001471909	PRKCA	3.659701811	8.382534028	-1.19566033	9.88E-32	7.98E-31	RPS2
ABCC5	1.069677029	1.011898866	1.130754254	0.017431552	SLC2A14	0.151614152	0.028615941	2.405513481	1.76E-19	4.70E-19	SUCLA2
MTAP	0.632954836	0.499907137	0.801412492	0.000145465	VNN2	3.860287269	1.094486039	1.81845466	5.05E-19	1.31E-18	ACAD8
B3GAT2	2.395017751	1.021304077	5.616456603	0.044596549	SLC2A1	63.5368224	16.14210251	1.976764438	6.96E-30	4.24E-29	NDUFS1
ARSG	0.73477783	0.555249735	0.972352486	0.031074807	PIP4K2C	12.32394639	32.97064292	-1.419717708	1.01E-40	2.59E-38	NNMT
ORMDL3	1.039625033	1.006254286	1.074102464	0.019568507	TBXAS1	5.04850144	1.432715124	1.817103435	8.93E-36	1.89E-34	TRIB3
B3GNT2	0.951902172	0.926378029	0.978129573	0.000378612	RPL18	147.9483398	68.24917944	1.116209902	1.91E-31	1.46E-30	MUT
ALDH3A2	0.960259819	0.94747628	0.973215836	3.02E-09	ABCA1	11.2231736	3.708622333	1.597525424	2.81E-31	2.09E-30	UQCRFS1
ECSIT	0.966443876	0.940250272	0.993367185	0.014905646	GYG2	0.542807894	1.261204424	-1.216288528	6.89E-24	2.44E-23	P4HB
ABCD1	1.130872313	1.072369793	1.192566405	5.68E-06	CAT	51.07843203	123.5289486	-1.274063028	2.08E-33	2.36E-32	ACADSB
NDUFV3	1.078099255	1.00208288	1.159882107	0.043827388	RPL22L1	9.217157221	3.563499847	1.371026997	1.91E-30	1.27E-29	RPS19
ADO	0.921440525	0.850135226	0.998726574	0.046483984	HPSE2	0.169838211	0.909455705	-2.420842295	3.26E-26	1.43E-25	PNP
B3GALNT1	0.933110699	0.898711571	0.968826489	0.000303274	DCN	6.456298469	37.8382284	-2.551065363	3.60E-34	4.99E-33	ACBD7
PLTP	1.002355707	1.001237895	1.003474768	3.58E-05	HIBCH	5.346094034	12.27786082	-1.199502102	3.47E-20	9.70E-20	TNFAIP8L2
MED22	0.851080435	0.757400862	0.956346822	0.006725258	ACAD11	1.668150987	0.706895113	1.238681803	4.26E-09	6.76E-09	DCN
PRKAA2	0.847793219	0.805508238	0.892297941	2.53E-10	SUCLG2	21.86739409	53.0390475	-1.278273561	1.34E-36	4.10E-35	HMGCR
ATP5S	0.843348977	0.72826231	0.976622692	0.022848426	LIPH	0.766007959	9.909148458	-3.693329798	9.04E-39	6.25E-37	PPARGC1A
NUP210	1.043863304	1.022967231	1.065186219	3.17E-05	SLC44A4	8.055449093	29.69985932	-1.882419171	3.52E-31	2.57E-30	ADA

HSD17B12	0.933944663	0.89044768	0.979566405	0.004978957	NUDT1	4.451537321	1.930520633	1.205313677	6.51E-33	6.59E-32	HK3
OSBPL8	0.92606751	0.889544679	0.964089893	0.000183053	AFMID	7.680142191	15.65068176	-1.027020578	9.13E-26	3.81E-25	ALDH6A1
PDSS2	0.867949944	0.800965184	0.940536644	0.000548294	SUOX	7.440068766	15.40417578	-1.04993363	6.43E-38	3.53E-36	RPL36A
NME1	1.025765376	1.013731792	1.037941805	2.39E-05	CKMT1A	0.235902842	0.518990773	-1.137516087	6.11E-28	2.95E-27	NADSYN1
IMPDH2	1.012231136	1.000096154	1.024513361	0.048201343	ALOX12B	0.354813187	0.090868373	1.965209486	1.93E-16	4.27E-16	ACADM
OAZ2	0.950322429	0.922447258	0.979039952	0.00079502	PSMB9	24.81906221	5.288747181	2.230450688	1.07E-37	4.97E-36	CAT
NUDT5	1.076706264	1.042597138	1.111931288	6.80E-06	LRAT	0.655736692	0.136894504	2.2600521	1.50E-18	3.78E-18	BMP1
UBB	0.996493357	0.994382343	0.998608852	0.001168037	EPM2A	1.354741728	3.451112939	-1.349043849	3.09E-39	2.73E-37	PFKP
MFSD2A	1.074551329	1.038060947	1.112324437	4.52E-05	RPS20	268.0015859	132.8400724	1.012551124	2.04E-28	1.07E-27	NNT
THRAP3	0.978423446	0.957827441	0.999462324	0.044482967	SLC2A3	22.7955221	5.570609986	2.032843235	1.51E-26	6.76E-26	PIP5K1B
RPL18	1.001752363	1.000159941	1.003347322	0.031006252	CYP17A1	3.803805891	13.60738757	-1.838874584	2.61E-32	2.40E-31	NUDT1
MED24	1.091212848	1.012447149	1.176106309	0.022396035	PLA2G6	2.340319577	0.954664944	1.293639158	3.84E-11	6.70E-11	CHST11
SLC25A14	1.43572317	1.162263874	1.773522404	0.000794335	SLC25A10	4.596092748	9.414016619	-1.034402488	9.34E-09	1.45E-08	CDS1
ABCA1	0.969674834	0.94308401	0.997015403	0.029956998	HSD17B3	0.735941346	0.084911115	3.115565469	5.09E-24	1.81E-23	PCCA
GYG2	1.204967965	1.121608765	1.294522511	3.44E-07	CHST1	3.989618213	1.472567306	1.437917118	1.68E-06	2.37E-06	GNG7
GNG10	0.907044572	0.84485824	0.97380817	0.007094075	ENTPD2	5.178703673	2.156894928	1.263635111	1.33E-11	2.36E-11	AUH
MED17	0.743828787	0.600824609	0.920869845	0.006593294	RPS19	194.8100333	72.31161458	1.429768688	2.36E-35	4.12E-34	RRM2
HADHA	0.98713509	0.980439901	0.993875999	0.000192189	CDO1	0.56376283	1.754360218	-1.637784737	9.38E-34	1.16E-32	ENPP3
CYP2U1	0.781144848	0.669847347	0.910934822	0.001635914	ADHFE1	2.038163023	4.49728644	-1.141785325	1.04E-24	4.04E-24	DARS
PIK3CB	0.927466904	0.888259578	0.968404821	0.000633643	PFKP	110.7615584	28.33611743	1.966743257	2.81E-33	3.00E-32	SLC25A4
CAT	0.981709717	0.974271175	0.989205052	1.97E-06	NDST3	0.034714623	0.282085663	-3.022517943	1.09E-37	4.97E-36	ACAA1
STS	0.941470487	0.890389649	0.995481785	0.034084458	HS3ST3B1	0.841999608	2.009527624	-1.254964943	1.52E-30	1.02E-29	SLC27A3
RPL6	1.00384596	1.00107078	1.006628834	0.006574683	CETP	5.432347509	2.257560383	1.266811196	2.15E-18	5.33E-18	UGT8
SERINC1	0.990461728	0.986926851	0.994009266	1.49E-07	CES3	14.38109304	3.94590223	1.865746218	3.96E-12	7.25E-12	HSD3B7
HADHB	0.978590996	0.967693429	0.989611284	0.000152037	LIPN	0.110910758	0.03640515	1.607184865	6.16E-12	1.11E-11	HIBADH
RPL22L1	1.032111075	1.021716305	1.0426116	9.37E-10	PLA2G1B	0.64208825	0.283904363	1.177366578	2.04E-05	2.71E-05	ABCC3
PNPLA4	0.890574927	0.837664019	0.946827944	0.000208622	ACOT6	0.127494455	0.542113838	-2.088161328	1.12E-09	1.83E-09	AK7
RPLP1	1.001481312	1.000730485	1.002232702	0.000109624	SPHK2	1.96998958	4.412089446	-1.16327404	1.29E-34	1.91E-33	RPL37
DCN	1.011107691	1.001764431	1.020538093	0.019693048	GSTO2	0.828437673	2.900973017	-1.80807181	1.97E-33	2.29E-32	ABCD1
HIBCH	0.82413689	0.765600101	0.887149326	2.67E-07	ST3GAL4	6.453408047	15.43631657	-1.258195382	7.10E-25	2.77E-24	RPL18
SMARCD3	1.100601118	1.049611413	1.154067883	7.48E-05	AANAT	0.173324435	0.026894143	2.688111157	2.01E-29	1.14E-28	PLIN2
CHST12	1.40746705	1.081705001	1.831334324	0.010937282	FBP1	25.50755822	122.671715	-2.265805934	4.77E-26	2.06E-25	ABCA1
ACAD11	0.685067364	0.590987401	0.794124024	5.21E-07	PTGS2	2.5058454	5.727144401	-1.19251857	2.04E-14	4.08E-14	SCD5
NDUFAF3	1.020795535	1.006371771	1.035426027	0.004586153	BMX	1.104408092	0.376925734	1.550921162	3.29E-19	8.58E-19	SLC44A4
BCKDHA	0.673574707	0.565281325	0.802614319	9.93E-06	PLA2R1	1.316313958	10.68592537	-3.021136309	1.84E-18	4.58E-18	HYAL1
STXBP1	0.956733189	0.930487819	0.983718837	0.001829356	PNPLA7	1.717209593	0.564264138	1.605623572	3.18E-16	6.97E-16	PRKD1
SUCLG2	0.963934916	0.943081059	0.985249905	0.000996183	ACLY	88.47434425	33.71824625	1.391729663	1.39E-30	9.40E-30	PLCB2
NAMPT	1.022698722	1.011133369	1.034396358	0.000109725	AUH	10.78687134	23.52458606	-1.124892856	2.04E-32	1.91E-31	CYP2J2

SLC44A4	0.954017001	0.930971869	0.977632589	0.000161193	CACNA2D2	0.340372415	1.648251247	-2.275750149	2.08E-39	2.07E-37	ACLY
IQGAP1	0.973779694	0.956996583	0.990857136	0.002740453	PIK3R5	2.584320304	0.361425004	2.838016668	1.00E-37	4.82E-36	HS3ST3B1
PSMA1	1.078796114	1.046324054	1.112275926	1.15E-06	TPO	0.018535719	0.004699489	1.979732136	9.09E-15	1.85E-14	CYP51A1
MGAM	0.91967733	0.890948123	0.949332929	2.33E-07	SLC45A2	0.260007386	0.05818203	2.159907077	3.97E-11	6.91E-11	L2HGDH
NUDT1	1.094316373	1.047949251	1.142735035	4.50E-05	PTGR1	20.62718435	51.32649625	-1.315156876	2.38E-33	2.63E-32	RPL22L1
ABHD4	1.031901904	1.004926732	1.059601167	0.020146609	ALDH3B2	0.052817701	0.780699234	-3.885673454	1.33E-39	1.63E-37	ATP5S
PGM2	0.898200228	0.849000201	0.950251423	0.000187448	CHST15	16.10054749	5.040470694	1.675479379	3.49E-23	1.16E-22	PHKG1
PIK3C3	0.723101717	0.616502063	0.848133566	6.77E-05	ENPP6	0.221967216	6.668230133	-4.908885381	4.54E-35	7.45E-34	PSMB10
ANKRD1	1.058084885	1.00822274	1.110412987	0.021878739	PLA2G3	0.026275728	0.115454612	-2.135523192	4.10E-43	4.20E-40	VKORC1
FFAR1	31.70579079	2.805906392	358.264685	0.005207381	MOGAT2	0.029481677	0.562337691	-4.253546258	3.16E-32	2.79E-31	NR1H3
CHKA	1.083394199	1.016027494	1.155227587	0.014469171	GLB1L	15.96044265	7.009834222	1.187048433	2.89E-19	7.58E-19	RPL28
CSNK2A2	1.059111993	1.009015458	1.111695768	0.02017958	TNFAIP8L2	6.368755729	1.337980286	2.25095468	2.21E-34	3.20E-33	AANAT
ADAL	0.729744561	0.554900229	0.959680852	0.024167924	SCD	90.01779866	11.16170549	3.0116528	3.02E-35	5.17E-34	HPGD
XYLB	0.668366884	0.543440752	0.822011029	0.000135337	SLCO1A2	0.075019757	0.362725052	-2.273533891	2.66E-26	1.17E-25	IYD
MBTPS2	0.886917982	0.791615269	0.993694205	0.038543302	DGAT2L6	0.018297977	0.00478398	1.935400751	8.27E-06	1.13E-05	RPL13
NDUFB6	0.960580527	0.940392865	0.981201563	0.000206334	CYP4B1	0.298382399	0.883420445	-1.565937787	6.80E-18	1.63E-17	GNA15
RPL30	1.003099907	1.000007641	1.006201734	0.049435675	ARG1	0.281505714	0.056402044	2.319344853	1.47E-12	2.73E-12	RPS20
DGAT1	1.141092896	1.08539229	1.199651969	2.35E-07	CAV1	67.82712973	14.6949154	2.206545389	5.37E-35	8.45E-34	TAZ
LPCAT3	0.948545789	0.910012557	0.988710658	0.012541281	ENTPD8	0.43740238	1.445470081	-1.724505773	2.62E-25	1.06E-24	FABP5
OSBP	0.931215518	0.903942875	0.959310998	2.61E-06	LDLR	2.576844187	6.403849768	-1.313334158	9.73E-21	2.80E-20	ENTPD1
TEAD3	1.076895712	1.032385304	1.123325148	0.000581986	GCDH	3.993787816	8.152330028	-1.02945477	1.44E-26	6.49E-26	PANK1
SLC19A1	1.452921642	1.305827586	1.616585007	6.90E-12	GPC2	0.165825005	0.041112318	2.012018952	1.09E-24	4.21E-24	GOT1
COQ7	0.756463854	0.671330988	0.852392594	4.61E-06	SCARB1	34.56733916	2.450124358	3.818482674	1.22E-39	1.62E-37	ACAT1
PAOX	0.892651119	0.810124263	0.983584934	0.021769368	CD44	19.1090518	7.405462972	1.367594356	2.19E-18	5.42E-18	ABCG1
CARM1	1.169129902	1.081995659	1.263281158	7.68E-05	PLA2G16	42.23984463	20.04850131	1.075110134	4.40E-28	2.19E-27	IL4I1
MED13	0.885431643	0.825290814	0.949955071	0.000697558	ALOX12	1.025930398	0.42439849	1.273441429	2.06E-24	7.64E-24	ARG2
SLC46A1	0.665352987	0.535755329	0.826299942	0.000227693	CREB3L3	8.002731616	1.220201108	2.713373581	2.74E-19	7.21E-19	GDPD5
PSMB9	1.009320559	1.000260567	1.018462613	0.043737783	CTRC	0.049785035	0.010162027	2.292523942	8.57E-18	2.04E-17	EPHX2
LRAT	1.209468758	1.025613854	1.426282095	0.023784706	PDK1	5.058250405	1.321960119	1.935959804	7.51E-35	1.16E-33	FBP1
EPM2A	0.440377949	0.316117337	0.613483399	1.24E-06	CIDEC	0.88057837	0.326088786	1.43318658	0.002331261	0.002774148	PARP10
RPS20	1.001478045	1.000596643	1.002360223	0.001010123	NR1H3	8.723172943	4.204288542	1.052991307	5.41E-30	3.37E-29	MTTP
ADCY9	0.824355059	0.770129894	0.88239824	2.64E-08	GOT2	25.76515548	55.53744931	-1.108039621	5.42E-35	8.45E-34	GNAI1
HSD11B1	1.015977762	1.005100305	1.026972937	0.003898213	HSD11B2	27.00716632	206.4807	-2.934592757	4.49E-38	2.55E-36	PARP6
HSPG2	0.982181068	0.97459363	0.989827576	5.52E-06	SLC10A1	0.168367841	0.019426891	3.115489645	8.92E-07	1.27E-06	PPARA
B4GALT7	1.044183274	1.014323485	1.074922079	0.003492313	GLTP	23.18606296	48.31644542	-1.059256453	1.83E-24	6.88E-24	GPC2
PLA2G6	1.169730001	1.104432842	1.238887709	8.83E-08	RPS8	350.7467918	174.3955486	1.008066692	1.59E-29	9.38E-29	HMMR
CYP2R1	1.197593026	1.021170877	1.404494672	0.026580765	G6PC2	0.06404543	0.024653915	1.377278893	0.003838089	0.004516568	ELOVL3
DMGDH	0.950136299	0.932665567	0.967934293	6.60E-08	ACAD8	3.498253484	7.28976025	-1.059236536	3.27E-36	7.66E-35	IDUA

PSMC3	1.015712746	1.003790624	1.027776469	0.00965316	SDC3	17.34777311	8.28777425	1.065693868	1.44E-23	4.96E-23	PSAT1
HSD17B3	1.17041013	1.089942872	1.256818048	1.49E-05	ADCY8	1.002621743	0.108427298	3.208977506	0.002645	0.003138103	HSD17B3
CHST1	1.028183828	1.005943729	1.050915626	0.012734474	PLA2G2D	1.17059791	0.10591699	3.466239676	2.11E-19	5.59E-19	GYG2
ENTPD2	0.947302322	0.90844688	0.987819661	0.011293861	AOX1	14.05488769	36.75379622	-1.386821345	5.09E-08	7.70E-08	CRYL1
GMPR2	0.943332986	0.917612726	0.969774172	3.53E-05	CYP39A1	1.254855272	3.964899986	-1.659763493	1.12E-31	8.86E-31	PLB1
RPS7	1.003128236	1.000456087	1.005807521	0.021732129	SCD5	9.379611275	30.30366839	-1.69189241	2.98E-31	2.19E-30	SDC3
RPS19	1.001256491	1.000425479	1.002088193	0.003035719	ASMT	0.063562388	0.014891983	2.093637436	2.93E-16	6.44E-16	LIPA
ADCY1	0.634439325	0.459334693	0.876296225	0.00575642	CYP2J2	52.21073534	1.437270426	5.182943038	1.17E-30	7.97E-30	CYP1B1
PLCB1	0.848927088	0.784792935	0.918302354	4.38E-05	HSD17B7	3.497367602	1.106739867	1.659953279	2.48E-32	2.29E-31	LPIN3
PNPLA2	1.019652447	1.001938477	1.037679594	0.029514382	CPNE7	1.211761063	0.244287468	2.310453496	3.78E-11	6.61E-11	GLIPR1
PON2	1.011125866	1.005212368	1.017074153	0.00021805	RAPGEF3	2.285044075	7.533435167	-1.721085876	1.67E-36	4.65E-35	ANGPTL3
SEC24C	0.932940029	0.895024813	0.972461417	0.001041255	TH	0.012485069	0.054438489	-2.124423228	1.02E-24	3.97E-24	DPEP1
CPNE3	0.939480299	0.9166715	0.962856632	6.41E-07	PDHB	9.711039788	21.86855007	-1.171159887	3.87E-37	1.47E-35	LCAT
PFKP	0.993709679	0.990374908	0.997055678	0.00023396	PTPN13	4.817310848	16.96199097	-1.816005596	6.83E-39	5.18E-37	CYP21A2
AMDHD1	0.804533377	0.712610141	0.908314263	0.00044235	ALPI	5.040047415	0.763874302	2.722030143	0.024883035	0.028157118	CHDH
DTYMK	1.072266255	1.037276427	1.108436375	3.75E-05	CHST6	0.055354491	0.374786808	-2.75929789	1.67E-16	3.73E-16	ETNK2
NDST3	14.52284076	2.346428032	89.88679844	0.004014459	SLC6A8	81.88765431	25.86596617	1.662590881	2.95E-24	1.06E-23	SLC16A8
HS3ST3B1	1.189633294	1.047091587	1.351579357	0.007661663	BCAT1	2.402357403	0.887504932	1.436623758	1.69E-17	3.92E-17	PLA2G4D
HACL1	0.85658843	0.753798146	0.97339552	0.017625439	PC	5.074872195	13.14827386	-1.373430016	6.36E-15	1.31E-14	HIBCH
GALE	1.027820855	1.002656649	1.05361662	0.030026074	MTHFD2	6.126585107	3.049140875	1.006680349	1.55E-17	3.62E-17	CHKB
PGS1	1.227187364	1.13931281	1.321839634	6.65E-08	SLC16A8	0.426491983	0.098107919	2.120077126	2.98E-21	8.83E-21	PHYH
PSMB5	1.011181142	1.000031323	1.022455276	0.049356305	RPS2	313.8427391	116.2507789	1.43280145	2.30E-36	5.89E-35	AKAP5
SLC25A16	1.100583703	1.015648882	1.192621297	0.019340445	ALOX5AP	8.71263291	3.809420556	1.193537191	2.67E-15	5.58E-15	BMX
PGK1	0.996295197	0.994226289	0.99836841	0.000466012	CTGF	83.9272039	190.8310664	-1.185085632	1.30E-19	3.51E-19	PCK1
MED10	1.047549563	1.017265131	1.078735576	0.001911675	PIP5K1B	1.041505637	3.304663321	-1.665832649	6.40E-33	6.52E-32	SPHK1
INPP5K	0.904512578	0.844516106	0.968771344	0.004157001	LPCAT1	42.07852589	7.316948347	2.523770185	3.13E-36	7.43E-35	TMEM86B
AANAT	3.547900458	2.037585011	6.177704291	7.63E-06	MOGAT3	1.956139507	0.397957991	2.297321212	1.01E-11	1.81E-11	LRAT
FBP1	0.981831232	0.972900697	0.990843743	8.39E-05	AWAT1	0.024828007	0.007011761	1.824119727	0.007036083	0.00816514	CD44
GNA11	0.921775747	0.884846804	0.960245914	9.44E-05	PYGL	16.08835989	3.956743111	2.023631952	1.28E-35	2.45E-34	ABCB4
AMY2A	57.98939589	5.575481216	603.1353896	0.000678539	CACNA1A	0.184013764	0.079909042	1.203383019	1.08E-15	2.30E-15	TREH
BMX	0.690763763	0.546687958	0.872809743	0.001936546	UPP1	8.786879672	4.363011333	1.010026717	1.52E-15	3.24E-15	LUM
HSD17B8	0.944430786	0.921127702	0.968323401	7.28E-06	HMMR	1.063885127	0.416237058	1.353865063	1.69E-24	6.39E-24	VCAN
PNPLA7	1.114570271	1.031521903	1.204304905	0.006041606	MCCC1	8.025507583	16.67542453	-1.055058945	4.19E-31	2.96E-30	CD38
BDH2	0.969887167	0.949819946	0.990378355	0.00415279	OXCT1	12.88907579	31.02574981	-1.267317258	4.91E-33	5.07E-32	MTHFD2
HMGCL	0.919075018	0.876768405	0.963423048	0.000448528	LCAT	4.805940794	1.370049468	1.81059089	4.20E-23	1.38E-22	BCAT1
RRM2B	0.971146509	0.945391533	0.997603118	0.032764684	PLA2G5	0.344077579	0.049894417	2.785783578	4.29E-26	1.85E-25	CHST6
CBR4	0.839101795	0.761380813	0.924756456	0.000404187	ACOT12	0.050829479	1.010256045	-4.312911721	1.76E-41	9.33E-39	B3GNT4
ACLY	0.990933611	0.986886351	0.994997469	1.29E-05	PSMB8	72.09384719	23.5900901	1.61169521	1.60E-39	1.70E-37	PNPLA7

SYNJ1	0.8054039	0.691125833	0.938577914	0.005573363	GIF	0.022848348	0.007729451	1.563651939	0.041069202	0.045597418 GPAT2
AUH	0.924586755	0.891321804	0.959093185	2.74E-05	LDHB	146.1951428	428.8609028	-1.552614419	8.55E-39	6.18E-37 ALOX5AP
PIK3R4	0.844597564	0.78533155	0.908336161	5.37E-06	SREBF2	11.93171555	28.61649194	-1.262045335	1.42E-35	2.69E-34 CBS
COX5B	1.001881617	1.000012204	1.003754524	0.048521643	ACOX2	3.173125389	11.18087994	-1.817057296	5.29E-11	9.17E-11 AMY2B
PSMA4	1.089478565	1.049387898	1.13110085	7.46E-06	PCK1	22.33713424	149.2913056	-2.740614137	3.76E-19	9.79E-19 SLC22A13
LPCAT4	1.208723672	1.135806744	1.286321747	2.35E-09	CRYM	3.93842758	8.631071331	-1.131919897	5.64E-07	8.13E-07 OSBPL6
FAM120B	0.891394126	0.824341307	0.963901094	0.003958641	BMP1	7.837272629	2.151834175	1.864784779	2.28E-33	2.54E-32 LPA
PARP14	1.019551605	1.000180084	1.039298313	0.047887886	SHMT2	46.41954134	11.77514142	1.978987881	1.22E-37	5.26E-36 CSAD
SLC45A2	1.063613154	1.006283076	1.124209447	0.029144938	LIPG	0.981768137	2.432585054	-1.309035998	2.55E-23	8.62E-23 RAPGEF4
IVD	0.956525846	0.92382566	0.990383504	0.012264634	SLC16A1	18.25550476	4.252015376	2.102112841	1.06E-35	2.10E-34 B3GAT2
SLC5A6	1.129154854	1.08017223	1.180358695	7.95E-08	HMGCR	5.441092878	11.052227	-1.022368739	4.56E-34	6.15E-33 ALDH1L2
MOCOS	1.177421657	1.118650351	1.239280672	4.06E-10	HSD3B7	31.08186301	6.777910042	2.197160585	9.71E-32	7.88E-31 ENTPD2
STARD3NL	0.956798637	0.921161276	0.993814715	0.022587835	GPD1	13.81676874	42.52470183	-1.62188086	3.88E-05	5.07E-05 PYCR1
SHMT1	0.970328311	0.954653719	0.986260266	0.000288986	NCAN	0.014829533	0.003707815	1.999832087	2.38E-07	3.49E-07 CYP4F3
STK11	1.139163596	1.034091445	1.254911937	0.008316815	ABCG1	12.12078588	5.000240292	1.277413911	5.52E-28	2.68E-27 SLC6A7
LCLAT1	0.718755059	0.590936001	0.874221293	0.000948271	CYP8B1	1.998815599	5.545651242	-1.472211508	8.43E-08	1.26E-07 CPNE7
TALDO1	1.009962716	1.003875504	1.016086838	0.001308984	DAO	1.382285341	12.55426448	-3.183050145	1.16E-11	2.08E-11 PLA2G6
TNFAIP8L2	1.052397479	1.020848945	1.084920996	0.001006368	RPL28	77.21067196	37.06324458	1.058811086	1.81E-29	1.05E-28 SLC45A2
NUP107	1.258060856	1.117316777	1.416533924	0.000149117	AHRR	0.572329816	0.206329636	1.471895713	2.48E-10	4.18E-10 ENPP2
MED15	1.099732851	1.058313615	1.142773112	1.21E-06	GDPD5	1.322098542	0.506761117	1.383451975	8.00E-27	3.65E-26 ACOX2
PPP2CA	0.974154984	0.950128317	0.998789234	0.039874646	AKR1D1	0.11510989	0.009702846	3.568460079	4.27E-06	5.90E-06 CPT1B
IDH3A	0.857875816	0.766260796	0.960444434	0.007805304	BCAN	0.616614545	0.156655338	1.976774987	5.38E-26	2.32E-25 HAO2
SLC10A2	0.940473637	0.909894589	0.972080362	0.000273711	ACSL6	0.181689791	0.78189695	-2.10550112	9.22E-34	1.15E-32 ALDH1L1
MBOAT7	1.061245403	1.044535433	1.07822269	2.12E-13	SLC5A4	1.068480623	0.295514144	1.854261656	5.39E-19	1.39E-18 PCK2
ACAT2	1.183241247	1.024010744	1.367231601	0.022505651	AGT	50.60252513	23.32483383	1.117342576	9.34E-09	1.45E-08 CH25H
ADIPOR1	0.979602611	0.961354502	0.9981971	0.031709433	AGXT	0.822009876	3.632917127	-2.143900824	1.16E-12	2.18E-12 UROC1
SLC44A3	0.94551812	0.923658278	0.96789531	2.68E-06	ACSL4	13.09191682	37.34497875	-1.512237936	2.99E-38	1.76E-36 KERA
KMO	1.024578632	1.004589608	1.044965391	0.015714137	PARP6	6.226735974	3.098685861	1.006819603	1.78E-25	7.24E-25 LPL
PIK3C2B	0.890166472	0.822583266	0.963302296	0.0038766	SLC16A3	31.64135822	3.891058964	3.023576782	2.24E-37	8.91E-36 HAS2
RPL4	1.002099087	1.000104896	1.004097254	0.039096686	FDFT1	11.39448641	23.19094181	-1.025225511	1.13E-37	4.98E-36 ACAD11
NDUFA10	0.930613216	0.873621793	0.991322521	0.025730094	RPL18A	79.90811203	37.21040181	1.102636	6.21E-30	3.80E-29 GK
LRP2	0.983016029	0.976296489	0.989781818	9.84E-07	CD36	16.4489814	3.561980571	2.207246696	2.55E-23	8.62E-23 GATM
GPC2	1.546309636	1.093803975	2.186016457	0.01360389	RPS14	183.6508046	88.95319097	1.045846952	1.08E-29	6.40E-29 AOX1
GPC6	0.973258222	0.950532136	0.996527662	0.024543983	UROC1	0.045588029	0.011053383	2.044167019	1.74E-09	2.82E-09 SARDH
CD44	1.009317547	1.00616766	1.012477295	6.04E-09	CYP27B1	0.492092382	5.799186388	-3.558849421	2.10E-19	5.58E-19 AGMAT
ESYT1	0.973555746	0.957268585	0.990120021	0.001849087	SUCLG1	19.33853684	64.30233056	-1.733392383	1.47E-36	4.25E-35 CYP4A22
LDHAL6B	25.23141553	4.552226975	139.8489867	0.000220215	TRIB3	18.04057482	2.151184735	3.06804198	9.85E-36	2.04E-34 CYP4A11
NUP54	0.904812085	0.830782797	0.985437966	0.021630877	AKR1C2	1.251670804	3.012281661	-1.267001499	0.000733587	0.000897107 CRYM

AGPAT3	0.95132373	0.920607838	0.983064451	0.002882629	KERA	0.06996501	0.002663817	4.71506696	1.83E-09	2.98E-09	SLC25A2
APRT	1.00848354	1.002256691	1.014749075	0.007511174	PARP10	16.35611095	8.04703	1.023301438	6.75E-26	2.88E-25	CHST1
PGLS	1.032752365	1.017635766	1.048093516	1.84E-05	DDC	20.87503853	63.70149566	-1.609548386	1.80E-05	2.40E-05	ADCY2
CIDEC	1.030121177	1.008689828	1.052007873	0.005664972	ARSH	0.005321176	0.040509803	-2.928454167	1.36E-19	3.65E-19	SLC5A1
SRD5A3	1.117441965	1.045820605	1.1939682	0.00101768	CYP51A1	0.812039224	1.791744011	-1.141743211	1.68E-30	1.13E-29	DDC
CDS2	0.851806455	0.800606222	0.906281037	3.95E-07	TCN1	0.681919131	0.076218819	3.161381508	0.000101671	0.000129927	CHST9
NR1H3	1.040970269	1.000277567	1.083318408	0.048426672	TYMS	12.37620179	2.853996469	2.11651317	1.43E-36	4.21E-35	AKR1C2
STARD7	0.976504359	0.95800919	0.995356593	0.01480904	CYP4A22	2.459057687	8.167694856	-1.731823389	1.63E-07	2.42E-07	SLC25A27
PSMB3	1.004055619	1.001083638	1.007036423	0.007449541	ABCB4	0.864833787	0.195752971	2.14338868	2.19E-18	5.42E-18	APOM
MTHFD1	0.909721763	0.866503263	0.955095868	0.000138955	SLC5A5	0.072965847	0.008798098	3.051957731	6.89E-18	1.65E-17	CIDEC
AP2M1	1.013372041	1.006556114	1.020234121	0.000114421	RPL10	282.6638575	137.9935456	1.034486638	8.93E-33	8.88E-32	ACHE
CPT1A	0.959184997	0.94042774	0.978316376	3.54E-05	ARNT2	4.725729053	16.22907947	-1.779972351	4.33E-34	5.89E-33	GNB3
HMGCLL1	0.171476131	0.05548106	0.529983808	0.002193054	SLC25A15	2.080018394	4.449283264	-1.096976665	1.30E-32	1.25E-31	AGXT2
CLOCK	0.840070227	0.748353023	0.943028177	0.003132571	GK	3.711115972	9.87483969	-1.411904242	1.11E-08	1.73E-08	SLC5A8
PSMD5	0.922192083	0.863939211	0.984372774	0.014971441	ADSSL1	13.95030413	2.430510576	2.520965258	4.48E-21	1.31E-20	BHMT
ACAD8	0.845298083	0.739959036	0.965632981	0.013325207	GRHL1	0.404817668	1.034578497	-1.353698949	3.77E-29	2.09E-28	
SDC3	1.020127743	1.002739897	1.0378171	0.023092401	FTCD	9.054115931	20.1618415	-1.15498173	7.69E-05	9.91E-05	
COX19	1.518073902	1.283099515	1.796079219	1.14E-06	ENPP2	70.05449092	23.147985	1.597590925	4.69E-11	8.15E-11	
MED8	1.115897557	1.055794164	1.179422468	0.000103619	GCGR	0.7829299	5.430422043	-2.79410928	2.20E-36	5.83E-35	
AIMP1	0.936923132	0.888423116	0.988070818	0.016284131	GLYCTK	3.875845214	7.931479182	-1.03307899	4.81E-06	6.61E-06	
BBOX1	0.991786464	0.988420312	0.99516408	1.99E-06	PFKFB1	0.141005244	0.298378802	-1.08139623	9.51E-19	2.42E-18	
SLC7A5	1.010845768	1.005893009	1.015822914	1.67E-05	CYP4F3	1.225016479	5.655357784	-2.206817142	3.06E-11	5.38E-11	
AOX1	0.983273607	0.9717739	0.9949094	0.004950444	ETNK2	6.529046657	20.55196175	-1.654331854	1.01E-21	3.09E-21	
MED29	0.965574675	0.933714421	0.998522066	0.040720935	NDUFS1	9.020242729	20.33792392	-1.172934256	8.93E-36	1.89E-34	
NUDT12	0.896003518	0.853128845	0.941032893	1.14E-05	PIK3CG	1.849939691	0.760539903	1.282382391	1.84E-14	3.70E-14	
PGK2	13755.92853	17.13266684	11044723.6	0.005230167	SLC6A7	0.021410828	0.005235733	2.031877154	3.70E-11	6.48E-11	
SCD5	0.963991712	0.943760904	0.984656195	0.000701868	PLB1	0.639511942	0.253432466	1.335369947	1.04E-23	3.64E-23	
CYP2J2	0.996236522	0.992780933	0.999704138	0.033430376	UPP2	0.480300644	9.156492904	-4.252785479	7.74E-29	4.19E-28	
CRAT	0.938383361	0.916822214	0.960451567	8.22E-08	APOC2	0.442778635	0.088198234	2.327763932	8.96E-16	1.91E-15	
GYS1	1.021197743	1.006974381	1.035622008	0.003376934	AGMAT	13.51153143	68.35658834	-2.338889192	5.88E-08	8.85E-08	
AACS	1.242918891	1.004240626	1.538323914	0.045623469	GPC5	0.074618444	4.265916853	-5.837179765	5.28E-43	4.20E-40	
CHPF2	1.077760076	1.051613863	1.104556362	2.28E-09	ACSM3	3.672314881	9.623633819	-1.389891982	4.57E-29	2.52E-28	
CPNE1	1.030028032	1.020373259	1.039774158	7.39E-10	SLC25A27	2.35259271	1.089425683	1.110683793	0.000760576	0.000929398	
EHHADH	0.972281603	0.963298925	0.981348045	2.92E-09	PRKAR2B	1.567726298	7.447831625	-2.24814675	3.09E-40	4.92E-38	
PSMD14	1.133191324	1.069456839	1.200724077	2.30E-05	CH25H	1.085744478	2.768572289	-1.350457577	1.26E-09	2.06E-09	
CPNE7	1.056911935	1.034006932	1.080324323	7.36E-07	HYAL1	11.76320343	24.93277394	-1.083762405	3.70E-31	2.68E-30	
RANBP2	0.900461399	0.852675352	0.950925495	0.000164136	AKR1C1	3.821344945	8.402919547	-1.136810178	1.97E-08	3.04E-08	
GNB1	0.980893784	0.971776541	0.990096566	5.15E-05	ADCY7	1.251110423	0.313740974	1.995563267	1.74E-24	6.57E-24	

NUP133	0.866668311	0.808176715	0.929393222	5.97E-05	ADRA2C	2.971283164	8.260074147	-1.475068631	2.05E-24	7.61E-24
G0S2	1.002536422	1.000829161	1.004246595	0.003579044	PANK1	2.990223588	7.949893347	-1.410682143	4.50E-28	2.24E-27
ACOT13	0.887912346	0.823182462	0.957732181	0.00208254	ABCC3	20.70616958	4.103354433	2.335185015	1.10E-31	8.76E-31
PISD	1.093391343	1.056991745	1.131044432	2.36E-07	SORD	3.156407983	13.67269904	-2.114942466	1.93E-24	7.22E-24
NUDT7	0.649715075	0.52996558	0.79652282	3.34E-05	SUCLA2	13.29967367	26.78668	-1.010124934	2.86E-36	6.90E-35
PTPN13	0.88470174	0.825233508	0.94845539	0.000559413	AK7	0.904119979	2.556986644	-1.499858486	1.27E-31	9.95E-31
EEF1E1	1.087185834	1.018187959	1.160859375	0.012463053	OSBPL6	0.160876948	0.397492202	-1.304968935	2.97E-14	5.86E-14
PIKFYVE	0.892213711	0.803746338	0.99041858	0.032300579	OGN	1.646940747	3.634924457	-1.142136731	6.32E-16	1.37E-15
AMPD2	1.142773315	1.082326066	1.206596505	1.49E-06	CYP1A1	0.110894701	0.93614846	-3.077546904	4.58E-28	2.27E-27
SLC25A1	1.005849497	1.000889499	1.010834075	0.020751238	ALDH1L1	7.277239312	15.76332322	-1.115108555	3.33E-10	5.57E-10
CHST6	2.946554373	1.996217144	4.349317759	5.35E-08	GOT1	40.84269312	84.95069431	-1.05654774	4.68E-28	2.30E-27
BCAT1	1.075268677	1.045938827	1.105420984	2.70E-07	CYP4A11	20.9747067	60.44897469	-1.527067232	2.57E-07	3.76E-07
MTHFD2	1.05982163	1.036273178	1.0839052	4.02E-07	GMPR	3.60522437	18.9570153	-2.394570883	3.39E-35	5.68E-34
RORA	0.717915334	0.639526488	0.805912556	1.94E-08	ENTPD1	7.055871642	3.469673056	1.024024591	3.97E-28	1.99E-27
UCP1	6.459306241	1.285196838	32.46400543	0.023540665	HK3	1.835402138	0.300916402	2.608661545	6.24E-34	8.00E-33
NR1H2	1.031320713	1.01016292	1.052921656	0.003544886	PCSK9	0.045161392	0.122465757	-1.439216544	2.35E-24	8.63E-24
TBL1X	0.946134865	0.918349716	0.974760668	0.000271695	HDC	0.349956859	0.093007408	1.911759548	1.06E-17	2.49E-17
SLC16A8	1.822884626	1.346179583	2.468399017	0.000103636	GLDC	3.381029429	16.88547622	-2.320248389	2.37E-36	5.89E-35
RPS2	1.00175654	1.000957311	1.002556407	1.64E-05	PPM1K	1.921652678	5.446468014	-1.502973353	2.00E-36	5.47E-35
ALOX5AP	1.022599519	1.008816063	1.036571299	0.001248018	CDS1	5.613819195	15.01953874	-1.419786002	1.22E-32	1.19E-31
TMEM126B	0.958966589	0.922218274	0.997179242	0.035583692	PIPOX	3.573386495	26.87580397	-2.910944044	8.50E-10	1.40E-09
PIP5K1B	0.778017016	0.625343023	0.96796551	0.024316655	APOC1	88.20676067	2.677430229	5.041968351	9.59E-41	2.59E-38
PDK2	0.911225907	0.875968683	0.947902213	3.88E-06	BGN	428.3880594	204.7638106	1.064957508	1.51E-11	2.69E-11
AGK	1.170317575	1.051737244	1.302267494	0.003909043	TAZ	5.38509528	2.512156556	1.100045496	2.19E-28	1.14E-27
ENTPD6	1.071419974	1.046596468	1.09683225	8.03E-09	GPC3	3.517973163	48.85896707	-3.795806982	1.90E-40	3.79E-38
HMMR	1.218289473	1.137250737	1.305102903	1.89E-08	OAT	14.82165811	33.61081736	-1.181218775	1.11E-36	3.53E-35
TK1	1.015196879	1.008090412	1.022353444	2.57E-05	CBS	0.026033986	0.055601049	-1.094715851	3.51E-15	7.31E-15
RAE1	1.338678916	1.167303631	1.535214312	3.00E-05	CPNE6	0.022183501	1.886353055	-6.409968829	2.26E-15	4.75E-15
PLEKHA2	0.973567497	0.955174413	0.992314764	0.005909704	IDH2	61.13909096	133.58927	-1.127637127	7.68E-26	3.25E-25
LCAT	1.063306344	1.029572711	1.09814525	0.000190162	PLD4	1.878126168	0.382278107	2.296599498	7.56E-29	4.11E-28
NCOA1	0.883616853	0.832790598	0.937545099	4.25E-05	EPHX2	14.85866984	37.34381275	-1.329564261	1.70E-26	7.48E-26
STX1A	1.140573073	1.083562597	1.200583093	4.97E-07	ALDOC	32.67437267	6.832748431	2.257621622	4.42E-25	1.74E-24
GGT1	0.988309064	0.980836127	0.995838937	0.002391784	CRYL1	64.89023218	133.7763104	-1.043749428	9.96E-24	3.49E-23
ACOX2	0.87782659	0.802913817	0.95972881	0.004194865	GPAT2	0.443956494	0.169482984	1.389277875	5.72E-16	1.24E-15
PCK1	0.994116846	0.988563216	0.999701676	0.03898448	ALOX5	12.6033022	2.980667235	2.08009456	3.05E-25	1.22E-24
CRYM	0.946574798	0.899246811	0.99639369	0.035903757	GM2A	28.08748678	10.97664703	1.35549011	5.97E-36	1.32E-34
MTF1	0.736909547	0.632154514	0.859023654	9.52E-05	PTGDS	15.73044518	37.2462633	-1.243536195	3.82E-14	7.51E-14
ACSS3	0.876967102	0.808231084	0.951548775	0.001618533	ALB	21.52170558	54.80645898	-1.348553511	5.03E-16	1.09E-15

ENTPD4	1.160001908	1.08261785	1.242917274	2.51E-05	TMEM86B	0.69513487	0.293071884	1.246038347	1.08E-18	2.74E-18
FDPS	0.939397818	0.907806614	0.972088379	0.000341063	PNP	15.98834704	43.28292014	-1.436777042	3.13E-35	5.30E-34
RFK	0.966301736	0.936211197	0.997359408	0.033688589	ALDH6A1	9.606474867	74.57451519	-2.956603663	7.95E-34	9.97E-33
INPP5F	0.823555059	0.72554092	0.934810038	0.002676181	BCKDHB	4.431452988	10.61940158	-1.260850757	1.10E-35	2.15E-34
BMP1	1.081634319	1.055125249	1.108809406	5.70E-10	GNG7	3.088619511	8.1336065	-1.39693304	1.68E-32	1.60E-31
FADS1	1.144876126	1.076329944	1.217787678	1.75E-05	ALDH1L2	0.878311024	0.352507251	1.317078976	4.36E-12	7.97E-12
HMGCR	0.821933789	0.752758544	0.897465938	1.23E-05	NNMT	268.4626517	18.31632946	3.87351907	9.25E-36	1.94E-34
CARNS1	2.999942424	1.718634456	5.236514672	0.000110963	RPL10L	0.028694667	0.009862353	1.540778896	3.39E-06	4.72E-06
HSD3B7	1.007250218	1.002809431	1.011710672	0.001353407	VCAN	25.42183894	7.009032463	1.858781183	6.47E-18	1.55E-17
PRKAR1A	0.979133283	0.968033996	0.990359832	0.0002886	SLC23A1	4.028049759	11.61591379	-1.527949241	1.75E-05	2.34E-05
SIN3B	1.077049101	1.017576286	1.139997838	0.010431862	PDP2	1.498487312	4.253299083	-1.505075438	1.43E-36	4.21E-35
CPT2	0.892807395	0.859211848	0.927716542	6.87E-09	TREH	0.972181992	4.600384207	-2.242456039	2.55E-18	6.26E-18
PSMD3	1.075425631	1.032728419	1.119888121	0.000434829	HIBADH	41.82693738	88.20252319	-1.076387561	1.03E-31	8.27E-31
ACOX3	1.184883697	1.063321393	1.320343392	0.002128807	HK2	17.0547609	1.589258743	3.423748599	2.37E-36	5.89E-35
ABCG1	0.935450639	0.904631829	0.967319378	9.47E-05	CHIA	0.023725193	0.053809764	-1.181448139	2.98E-22	9.44E-22
PTGR2	0.769098022	0.651920242	0.907337629	0.001852123	PNPLA3	0.381052559	1.083143219	-1.507162107	0.002809059	0.003327783
ORMDL1	1.11402944	1.068423926	1.161581618	4.12E-07	IL4I1	6.66819712	1.567318124	2.088998715	2.42E-27	1.13E-26
NT5C	1.020717786	1.001620091	1.040179613	0.033341467	MTTP	0.655611941	2.230482404	-1.766441731	7.28E-26	3.10E-25
CIDEA	1.381943355	1.010335498	1.890230957	0.042938419	APOM	16.86765048	38.40334376	-1.186972899	0.00079868	0.000974463
RPL28	1.002752744	1.000784798	1.00472456	0.006094493	PCCB	3.964180799	12.71099814	-1.680982655	7.18E-37	2.40E-35
GDPD5	1.388410093	1.190421017	1.619328421	2.91E-05	FABP7	84.19427879	1.387057525	5.923622676	5.27E-37	1.90E-35
MMADHC	0.973240197	0.958294297	0.988419198	0.000592162	NMNAT2	0.783576293	0.24856373	1.656457955	0.000557802	0.00068583
B3GNT7	1.161340601	1.028012114	1.311961185	0.016217322	MBOAT2	2.229418146	6.588774389	-1.563342896	8.02E-36	1.75E-34
CYP46A1	2.375053926	1.328767595	4.245197711	0.003508735	HAS2	0.406326596	0.835206191	-1.039492608	3.41E-09	5.44E-09
GPC1	1.053520339	1.029992845	1.077585257	6.05E-06	L2HGDH	1.803637072	4.625866342	-1.358814514	1.76E-30	1.18E-29
ADCY5	0.930804325	0.900931846	0.961667296	1.64E-05	HS3ST6	0.035730731	0.425850615	-3.575110095	1.99E-33	2.29E-32
PARP6	1.117796246	1.066005411	1.172103287	4.21E-06	P4HB	201.3146886	77.15507111	1.383619552	1.51E-35	2.80E-34
SARS	0.974874264	0.956847879	0.993240255	0.007534838	SLC5A3	10.00933542	38.45969168	-1.942001008	8.73E-26	3.65E-25
GART	1.089985401	1.000641474	1.187306549	0.048306726	STAR	0.05432004	0.024242841	1.163925792	9.68E-05	0.000123864
FDFT1	0.93175418	0.890563333	0.974850209	0.002183286	PITPNM3	0.306625001	0.833350431	-1.442447954	1.96E-27	9.28E-27
PITPNM2	1.104680315	1.016390519	1.200639494	0.019155168	CSAD	4.768674093	1.590289622	1.584298656	1.24E-13	2.40E-13
INPPL1	1.055780872	1.022279875	1.090379725	0.000969151	CHST11	7.156856489	2.084835428	1.779392546	1.13E-32	1.11E-31
EPRS	0.977563644	0.959417519	0.99605298	0.017613062	BHMT	71.68400267	162.9281698	-1.184512962	0.006948433	0.008069311
PRKACB	0.910733665	0.86805845	0.955506865	0.000134145	DEGS2	0.794410569	6.733143993	-3.083323596	9.17E-30	5.47E-29
OGDH	0.980967465	0.971557467	0.990468604	9.33E-05	CYP4F2	0.40139887	12.57277723	-4.969122998	4.28E-17	9.75E-17
IDH1	1.021732051	1.007105724	1.036570798	0.003472941	PYCR1	3.393914528	1.066275435	1.670370077	2.67E-11	4.71E-11
UROC1	5.906871531	1.862881968	18.72965217	0.00255632	GCAT	4.068903248	8.686227681	-1.094089793	3.77E-23	1.25E-22
SEPSECS	0.857892255	0.791942937	0.929333525	0.000172844	TPTE2	0.072295325	0.026987509	1.421610536	0.002995601	0.003540862

AP2A2	0.928643215	0.875491036	0.985022331	0.013824377	ITPR2	4.310921658	9.920941875	-1.202480749	7.03E-33	7.03E-32
PLEKHA5	1.054534951	1.012413437	1.098408932	0.010675222	NADSYN1	4.211279179	1.993024972	1.079298732	1.29E-33	1.53E-32
TRIB3	1.012405074	1.006711999	1.018130344	1.83E-05	HS6ST1	7.272424258	33.10929818	-2.186728159	2.30E-36	5.89E-35
VAPB	0.840213615	0.775670676	0.910127121	1.96E-05	DBT	2.868898877	8.216476389	-1.518022714	1.14E-40	2.59E-38
NUP160	0.881831106	0.824930526	0.942656471	0.000219716	GPT2	3.420571386	7.45080825	-1.123159596	2.93E-18	7.17E-18
GNG13	1.852288196	1.123216451	3.054595181	0.015724831	SPHK1	1.930841201	0.60873218	1.665349977	6.24E-19	1.59E-18
CHRM3	0.446022022	0.309931691	0.641869323	1.38E-05	HPD	3.977336003	114.4395138	-4.846639018	1.39E-24	5.31E-24
NMNAT1	0.639580668	0.521090026	0.785014892	1.91E-05	PHKG1	0.494667456	0.124915627	1.985505018	4.45E-30	2.86E-29
NUPL2	1.115553405	1.018741098	1.221565913	0.018234024	ADA	4.739863256	1.034734726	2.195584485	5.15E-34	6.77E-33
AKR1C2	1.04633238	1.001295329	1.093395143	0.043629695	AKAP5	0.567259281	1.415611447	-1.319345121	2.16E-19	5.69E-19
KERA	1.129789017	1.034115863	1.234313551	0.006870809	INPP5J	3.056462871	12.69138305	-2.05391435	3.99E-36	9.07E-35
HYAL2	0.972507788	0.955922036	0.989381311	0.001491529	CHST13	6.216933418	1.531017755	2.021712114	5.29E-22	1.63E-21
PARP10	1.027771224	1.007099932	1.048866806	0.008231025	GLIPR1	5.421736673	1.906767725	1.507625934	2.59E-23	8.72E-23
MMAA	0.658094543	0.55746494	0.776889085	7.75E-07	SLC25A5	143.5702395	313.8113806	-1.128140948	3.48E-33	3.67E-32
DDC	0.981238272	0.97174107	0.990828294	0.000135211	PFKFB4	5.080213919	0.961428575	2.40163766	2.61E-34	3.67E-33
FITM2	0.862687446	0.803473085	0.926265788	4.68E-05	CYP21A2	0.658639805	0.115831797	2.507458363	3.12E-22	9.86E-22
CYP51A1	0.371061482	0.251994201	0.546388062	5.13E-07	PFKFB3	41.65925882	101.8757353	-1.290101396	0.039439136	0.043879487
TMLHE	0.694887431	0.590182186	0.818168615	1.25E-05	GAPDH	1428.527476	522.868625	1.450008377	7.25E-37	2.40E-35
TYMS	0.96709141	0.941397	0.993487121	0.014868954	UST	1.337158139	3.268293092	-1.289367271	1.15E-28	6.16E-28
GLA	1.037079882	1.007658709	1.067360082	0.013154551	SLC25A2	0.052481912	0.016007136	1.71310506	6.79E-07	9.73E-07
ALDH18A1	1.021717873	1.007994682	1.035627896	0.001844989	SARDH	2.202010597	4.996210983	-1.182012989	5.11E-08	7.73E-08
GLCE	0.86889668	0.807236667	0.935266534	0.000182589	PDHA1	17.02476874	38.48976486	-1.176839657	1.81E-35	3.23E-34
CREBBP	0.8643869	0.805693188	0.927356373	4.86E-05	TYMP	33.13173079	4.304598149	2.944263104	4.69E-37	1.73E-35
AKR1B10	1.003754761	1.001253751	1.006262019	0.003236519	B4GALNT1	2.863771179	0.19358754	3.886858222	2.96E-28	1.51E-27
CYP4A22	0.951554149	0.911460171	0.993411812	0.02376483	IYD	0.238376528	4.423878008	-4.213997512	3.37E-29	1.89E-28
PPP1CB	0.97368071	0.960898173	0.98663329	7.63E-05	GNB3	0.239588608	0.086716699	1.466177562	0.004864243	0.005694636
ABCB4	0.679679161	0.53457923	0.864163319	0.001624215	GATM	49.7876089	209.6253164	-2.073954326	4.09E-08	6.22E-08
ENO3	1.02970071	1.006595542	1.053336229	0.011480941	IDUA	3.599673327	1.524896753	1.239154422	3.72E-24	1.34E-23
PIK3R1	0.967456265	0.93736586	0.998512603	0.040140777	TST	18.93979191	39.88242282	-1.074332575	1.85E-16	4.10E-16
NPAS2	1.271886498	1.158589959	1.396262114	4.36E-07	ARG2	9.539183227	37.12929571	-1.960620301	2.69E-27	1.26E-26
AGPAT5	0.955612819	0.923380963	0.988969771	0.009499043	UQCRFS1	15.40110064	37.26782847	-1.274897301	1.48E-35	2.78E-34
COX11	0.878004588	0.81084277	0.950729395	0.001353475	ACOT11	1.04262638	4.084562972	-1.969959458	1.24E-36	3.86E-35
DBH	1.865425055	1.495140659	2.327413553	3.34E-08	PLCB2	3.622450668	0.808865414	2.162994463	4.12E-31	2.94E-30
GK	0.917608574	0.852948991	0.987169812	0.021092161	PHKA2	16.81318925	3.605396111	2.221363735	1.61E-35	2.94E-34
AP2A1	1.113941039	1.064752082	1.165402406	2.83E-06	ALDOB	63.12515953	1633.284657	-4.693417314	3.15E-20	8.79E-20
DDHD2	0.875075295	0.802975987	0.953648407	0.002351815	ACHE	1.519225254	0.622196647	1.287893266	0.003934003	0.004622599
ETHE1	1.052383255	1.025256066	1.080228202	0.000127144	SLC5A1	11.15656198	2.409237749	2.211243839	1.46E-05	1.96E-05
PLEKHA1	0.906053251	0.853100053	0.962293333	0.001323187	CD38	1.385397938	0.36920732	1.907797369	7.46E-18	1.78E-17

SDHD	0.976517266	0.96210574	0.991144664	0.001733207	TDO2	0.619882968	0.149940492	2.047605828	3.02E-08	4.61E-08
ENPP2	0.997395324	0.994940263	0.999856443	0.038066316	CEL	0.537627863	3.982085128	-2.888844249	5.23E-35	8.45E-34
PIK3R2	1.477698759	1.037514245	2.104639655	0.030459587	PFKFB2	2.444758198	8.036807147	-1.716930683	9.19E-35	1.38E-33
CYP4F3	1.05031824	1.015661807	1.08615722	0.004134046	MUT	13.38728595	27.66262458	-1.047074535	1.02E-35	2.08E-34
ETNK2	0.937242709	0.903396039	0.97235748	0.000552964	SLC25A4	12.12265987	27.37052386	-1.174916768	7.24E-32	6.16E-31
KIAA1429	0.865857512	0.790199717	0.948759174	0.002018566	ENTPD3	0.111807125	1.0429266	-3.221553591	9.12E-38	4.68E-36
NDUFS1	0.930568711	0.88775156	0.975450976	0.002751847	RPL35	242.1192943	115.7821596	1.06430508	5.51E-26	2.36E-25
SLC6A7	14.62044319	1.023068923	208.9373985	0.048067502	NNT	12.24080903	29.40260222	-1.264244931	2.96E-33	3.14E-32
PLB1	1.956027461	1.484558312	2.577226772	1.86E-06	DPEP3	0.106191369	0.021403368	2.31075676	4.93E-21	1.44E-20
PNPO	0.959757209	0.927381121	0.993263588	0.018975087	PCK2	10.95643502	50.91182556	-2.216222344	1.11E-09	1.82E-09
RPL12	1.002179353	1.00092085	1.003439439	0.000684694	DPEP1	4.305901897	84.10286769	-4.287767638	3.23E-23	1.08E-22
ACOT4	0.883098003	0.827551214	0.942373196	0.000176385	PPARGC1A	4.334165749	14.43103276	-1.735348319	4.60E-34	6.15E-33
PDK4	0.997596736	0.996363088	0.99883191	0.000138266	PPP1R3C	29.83253044	7.713945653	1.951345467	1.92E-32	1.81E-31
AGMAT	0.969253394	0.954057942	0.984690866	0.000107274	SNAP25	2.606078364	0.645310559	2.013814929	2.12E-19	5.59E-19
OSBPL9	0.914885507	0.862943216	0.969954309	0.002855213	DPYS	14.36359881	36.99005904	-1.36472034	0.002107752	0.002511935
GALNS	1.114329677	1.04212618	1.191535777	0.001539041	VKORC1	29.04751399	13.38587731	1.117703001	5.19E-30	3.25E-29
FMOD	1.016075661	1.007815414	1.024403611	0.000128547	ABCG8	0.094230601	0.245624466	-1.38218672	2.17E-23	7.38E-23
D2HGDH	1.046479261	1.011753439	1.082396957	0.008324643	FBP2	0.042462518	0.117724905	-1.471157742	5.94E-24	2.12E-23
CUBN	0.975890578	0.968462577	0.983375552	3.84E-10	GPX2	0.833502518	1.981453477	-1.249300632	4.76E-28	2.33E-27
STARD3	1.192878397	1.095183545	1.299288031	5.22E-05	SMOX	7.1817972	3.338215635	1.105267765	2.10E-20	5.94E-20
IMPDH1	1.106300099	1.080837932	1.132362098	1.84E-17	NT5C1A	0.029656896	0.516031631	-4.121020002	3.76E-23	1.24E-22
A2M	0.997002286	0.995663332	0.998343042	1.19E-05	SULT2A1	0.369390249	0.018168638	4.345623584	0.001983486	0.002369164
SLC25A27	1.068225197	1.030987691	1.106807657	0.000266647	PLA2G10	0.130276026	0.051208102	1.34712762	5.08E-11	8.82E-11
FLAD1	1.178663971	1.108313765	1.253479656	1.65E-07	FOLH1	7.396661172	3.650400113	1.018819587	2.73E-11	4.80E-11
FAR2	0.837410729	0.733150536	0.956497601	0.008907558	AMT	3.817660885	7.996424444	-1.066666095	2.89E-28	1.49E-27
OSBPL7	1.412101415	1.26214972	1.579868357	1.69E-09	CBR1	27.86296553	56.00014792	-1.007081822	1.83E-29	1.05E-28
PRKAR2B	1.142556004	1.019220998	1.280815667	0.022217341	HPGD	2.548996387	10.93876474	-2.101448597	1.99E-29	1.14E-28
SCP2	0.913679873	0.88766562	0.940456509	9.04E-10	GNA15	2.909155838	0.868479335	1.744037155	1.10E-28	5.89E-28
CH25H	1.101758451	1.050570107	1.155440913	6.54E-05	BAAT	4.417637677	0.092684061	5.574810024	2.55E-11	4.49E-11
HYAL1	0.932077961	0.902366736	0.962767454	2.09E-05	ANGPTL4	340.8253171	11.07169999	4.944083844	1.58E-36	4.48E-35
SDHB	0.95095465	0.929337783	0.973074337	1.82E-05	CKM	0.129967094	0.902833927	-2.796314232	1.68E-16	3.74E-16
PSMC5	1.01437357	1.003145441	1.025727374	0.011972236	ACAT1	28.64223753	65.91870276	-1.202543651	4.68E-28	2.30E-27
SQLE	1.0748773	1.019345327	1.133434549	0.007632298	ADCY2	0.351482859	0.056779558	2.630010804	2.85E-06	3.99E-06
TCN2	0.992556389	0.988360988	0.996769599	0.000545967	PLA1A	23.00606604	7.496634472	1.617699344	1.25E-24	4.81E-24
PPARG	0.88293301	0.819572005	0.951192446	0.001049235	CPT1B	0.680381837	0.184735431	1.880884035	2.94E-10	4.95E-10
PANK1	0.78269037	0.7152848	0.856447972	9.69E-08	PIK3C2G	0.209681767	3.18925628	-3.926946714	2.84E-40	4.92E-38
ABCC3	1.016549229	1.005388666	1.027833682	0.003567185	HRASLS2	1.324025005	5.213910625	-1.977435484	6.31E-09	9.92E-09
SECISBP2	0.896709457	0.823635433	0.976266706	0.011944188	ALAS1	13.93552561	28.54401931	-1.034421078	1.16E-33	1.40E-32

SCO2	1.03775007	1.020080119	1.055726103	2.35E-05	RARRES3	80.1438001	34.38991556	1.220605345	1.57E-15	3.32E-15
SUCLA2	0.918499078	0.88840835	0.94960899	5.66E-07	ANGPTL3	3.314820816	11.02284145	-1.733493375	2.96E-23	9.90E-23
SMPD3	1.840184639	1.477073801	2.292559454	5.38E-08	LUM	28.76824695	71.24277354	-1.308266373	4.92E-18	1.19E-17
AK7	0.595548282	0.434044491	0.817146084	0.001322114	SLC27A3	6.898973392	2.442062149	1.49828178	8.14E-32	6.78E-31
FAAH2	0.840328622	0.768188722	0.919243114	0.000145473	ELOVL2	1.400430385	0.148109133	3.24113776	3.88E-31	2.80E-30
PRKD3	0.912525485	0.845077901	0.985356214	0.019464947	DARS	51.16707017	24.29280306	1.07468666	7.12E-32	6.09E-31
PECR	0.923931667	0.893257489	0.955659186	4.37E-06	HMGCS2	17.51671484	108.9225706	-2.6364988	2.58E-31	1.94E-30
OSBPL6	1.47851703	1.121059124	1.949953006	0.005619168	CSPG4	19.60701756	2.881595844	2.766430192	1.87E-33	2.21E-32
GPD2	0.891521181	0.81947601	0.969900285	0.007566402	CROT	3.563531557	8.556267792	-1.263673936	2.54E-39	2.37E-37
MAN2B1	1.022846874	1.003240472	1.042836444	0.02216176	CHDH	6.838296663	16.15601893	-1.240362824	8.88E-22	2.72E-21
TKT	1.031520528	1.018678916	1.044524024	1.20E-06	LDHA	283.8685387	90.01795639	1.656938245	7.71E-35	1.18E-33
COX16	0.925052533	0.86320561	0.991330662	0.02734298	HS6ST2	0.1425965	10.61975078	-6.218667533	5.22E-41	2.08E-38
CYP27A1	0.986826991	0.976710695	0.997048067	0.011659693	FABP1	1.126210216	26.40938491	-4.551502653	3.06E-18	7.46E-18
AHCYL1	0.97580312	0.958585649	0.99332984	0.007000969	TM7SF2	5.460770678	11.3594711	-1.056719186	4.95E-29	2.71E-28
ALDH1L1	0.938732838	0.911031623	0.967276347	3.52E-05	RBP2	0.139397015	2.037824387	-3.869758154	4.08E-36	9.14E-35
GOT1	0.987399699	0.979730617	0.995128813	0.001435559	HAO1	0.102745325	0.400816922	-1.963870664	2.43E-15	5.09E-15
PIK3C2A	0.9042045	0.865725806	0.944393446	5.67E-06	SGPP1	9.93914416	23.75221722	-1.256868658	2.51E-38	1.54E-36
CYP4A11	0.990625135	0.984636893	0.996649796	0.00232878	FOLR2	17.41474502	6.466904875	1.429162055	1.85E-16	4.10E-16
SGMS2	0.886796852	0.841077033	0.935001939	8.65E-06	PCSK6	7.786660459	0.974560968	2.99818037	2.09E-28	1.09E-27
PRSS3	1.040217411	1.025020356	1.055639779	1.51E-07	LIPA	44.87582433	19.79203097	1.181018777	1.51E-23	5.21E-23
ENTPD1	0.933396337	0.878301157	0.991947597	0.026390198	G6PC	2.819185278	29.90980479	-3.407268294	4.61E-13	8.72E-13
DSE	1.143717488	1.070219421	1.222263086	7.42E-05	PSMB10	18.93288573	7.361736236	1.362776357	4.98E-30	3.16E-29
HNMT	0.958568802	0.940723437	0.97675269	1.02E-05	PNMT	0.116488373	0.705595881	-2.598656181	8.69E-32	7.12E-31
PSME2	1.024193227	1.015537175	1.032923061	3.38E-08	MARCKS	48.25415192	22.78024789	1.082869635	1.56E-26	6.93E-26
MTHFR	0.829082755	0.756496805	0.908633335	6.08E-05	QPRT	17.63692326	39.26529278	-1.154655748	7.63E-22	2.35E-21
NSDHL	0.925558494	0.87115638	0.983357921	0.012316001	CHKB	1.851868177	0.561143344	1.722540145	7.36E-20	2.02E-19
SMPD2	1.117423669	1.058719036	1.179383399	5.52E-05	PIK3R6	1.443178438	0.137901625	3.387538325	3.59E-39	2.85E-37
ASNS	1.074368718	1.04579597	1.103722116	1.83E-07	PRKD1	4.184863745	8.566402708	-1.033508849	3.98E-31	2.85E-30
HK3	1.144450478	1.081791776	1.210738449	2.65E-06	RRM2	2.89982108	0.665280767	2.123928657	2.81E-32	2.51E-31
NCOR1	0.918928683	0.867579089	0.973317518	0.003954149	NEU4	0.186410772	0.574721458	-1.624377687	2.46E-21	7.40E-21
CDS1	0.884190098	0.834648422	0.936672387	2.87E-05	DCXR	14.67242986	64.1071605	-2.127377698	6.79E-33	6.84E-32
AGPAT2	1.009881261	1.003154911	1.016652711	0.003929169	CTH	1.53365613	5.554299703	-1.856629981	1.54E-37	6.44E-36
PTEN	0.920941223	0.876756471	0.967352696	0.001026663	GPHN	3.526872305	7.626959444	-1.112718686	2.94E-28	1.50E-27
TAZ	1.146949675	1.09514794	1.201201689	6.08E-09	SLC19A2	4.131322657	9.186015076	-1.152835412	1.70E-29	9.93E-29
MOCS3	0.665196856	0.491254357	0.900728616	0.008389298	KCNG2	0.036849303	0.096935331	-1.395385291	8.36E-09	1.31E-08
NDUFS4	0.973599747	0.960006368	0.987385603	0.000191822	PPARA	4.296827626	8.637702278	-1.007375689	5.64E-25	2.22E-24
NADK	1.117658157	1.035102229	1.206798441	0.004494933	RPL36	159.0903759	68.96026042	1.206009436	1.69E-31	1.30E-30
CBS	10.65171179	3.254209987	34.86528663	9.22E-05	ACBD7	0.114927291	0.359549795	-1.645470167	7.84E-35	1.19E-33

PSMA3	1.038047801	1.008659407	1.068292457	0.010822529	TYRP1	0.141356967	5.27942938	-5.222967112	1.50E-39	1.70E-37
RPS24	1.002441653	1.000516273	1.004370737	0.012913269	PHYH	16.68414104	37.20635557	-1.157071669	1.16E-19	3.14E-19
MOCS2	0.878451112	0.824411966	0.936032454	6.32E-05	ALDOA	333.2219371	158.1132872	1.07552477	2.79E-32	2.51E-31
EPHX2	0.959211652	0.940462175	0.978334927	3.55E-05	LTC4S	0.057682143	0.025926472	1.153698837	1.72E-07	2.54E-07
ACO2	0.979060823	0.970183619	0.988019254	5.27E-06	SLC22A13	0.62710593	4.29512116	-2.775917762	2.87E-14	5.67E-14
RAB14	0.958742724	0.941388277	0.976417099	6.17E-06	LCT	0.015330697	0.002808337	2.448635118	9.06E-09	1.42E-08
PRKAR2A	0.867311161	0.805227606	0.934181397	0.000172217	CHST9	1.996529543	0.848224842	1.234975779	0.000435715	0.000540314
CRYL1	0.98347576	0.977831938	0.989152158	1.39E-08	HAL	0.203634116	0.041285662	2.302266539	3.75E-16	8.18E-16
MBTPS1	0.906970617	0.864697953	0.951309872	6.08E-05	ADK	5.263814317	11.93990931	-1.181611375	3.17E-37	1.23E-35
FYN	0.947139151	0.913687124	0.981815928	0.003073883	ENPP3	78.62046653	3.092035128	4.66827631	3.06E-32	2.72E-31
LHB	2.560988002	1.923490092	3.409770381	1.20E-10	ECHS1	91.84253814	227.5192168	-1.308753986	2.96E-25	1.19E-24
GPAT2	2.006613875	1.463551095	2.751184606	1.52E-05	ACAA1	6.799000942	20.18380894	-1.56980378	7.74E-32	6.52E-31
GCLM	0.920635929	0.86633526	0.978340088	0.007676972	DEGS1	58.28496078	24.54561431	1.2476584	3.85E-31	2.79E-30
ENTPD5	0.951877234	0.916609571	0.988501864	0.010457279	AMY2B	1.425070527	0.407151139	1.807396977	2.31E-14	4.58E-14
PLD2	1.077855463	1.003964919	1.157184258	0.038529706	SLC5A2	0.329811965	8.089921186	-4.616410003	1.19E-19	3.23E-19
LDLRAP1	1.15936375	1.023953336	1.31268121	0.019621541	B3GNT4	1.644622184	0.467202585	1.815636032	2.06E-16	4.55E-16
RAP1A	0.944952177	0.908385343	0.982990999	0.004924362	CHSY3	1.513704245	0.327480898	2.20860069	2.65E-32	2.41E-31
TMEM86B	1.367630318	1.208431149	1.547802445	7.11E-07	FDX1	7.663216499	16.71874568	-1.125444644	9.64E-38	4.79E-36
PNP	0.963437104	0.938182426	0.989371606	0.005988897	LPA	0.039139077	0.165093796	-2.076604282	6.62E-14	1.29E-13
PI4KA	0.927978314	0.861406144	0.99969539	0.049069744	DIO1	1.008427804	26.5740953	-4.71984087	2.88E-21	8.59E-21
GBA	0.972957617	0.949477673	0.997018203	0.027838211	GADL1	0.022603521	0.482559229	-4.416086618	3.59E-36	8.29E-35
ALDH6A1	0.921040108	0.893389313	0.949546708	1.23E-07	HS3ST2	2.57976382	0.088621338	4.863441071	6.44E-37	2.23E-35
G6PC3	1.029039905	1.007848773	1.050676604	0.007009932	RPLP0	331.219276	162.7625281	1.025018042	5.91E-30	3.65E-29
SLC44A2	0.979665232	0.96975233	0.989679463	7.52E-05	CYP1B1	10.32415432	23.12736461	-1.163577267	1.52E-23	5.23E-23
OAZ1	1.002386989	1.0001549	1.00462406	0.036069637	ENPP1	1.355302349	2.860093124	-1.077447389	5.50E-23	1.80E-22
SGPP2	0.97512639	0.957820313	0.992745157	0.005834811	UQCRC1	39.0218911	83.75180597	-1.101836604	2.77E-32	2.50E-31
GNG7	0.784900002	0.698661804	0.881782875	4.53E-05	PAH	5.445535652	37.24339022	-2.773838534	5.88E-12	1.07E-11
SLC25A13	0.920801904	0.881493787	0.961862873	0.000209878	GNGT1	0.613884308	0.128110243	2.260580965	2.64E-05	3.48E-05
LIAS	0.847513139	0.761403975	0.943360614	0.002473429	GNAI1	10.08993414	23.59326063	-1.225458057	1.09E-25	4.52E-25
ALDH1L2	1.208643136	1.129270863	1.293594193	4.56E-08	PLA2G4F	1.057549357	6.118355944	-2.532419044	2.09E-36	5.63E-35
SLC27A2	0.955269048	0.940890623	0.9698672	3.34E-09	ACADM	17.04715657	47.14106	-1.467453078	2.02E-33	2.32E-32
NNMT	1.001070903	1.000433165	1.001709048	0.000995037	PCYT2	3.660777587	9.196652833	-1.328958756	1.14E-30	7.81E-30
GNG12	0.974442423	0.96252112	0.986511376	3.75E-05	INPP5D	6.462947246	1.973086658	1.711737895	7.42E-34	9.37E-33
AAAS	1.144269563	1.100716039	1.189546429	9.99E-12	IDO1	12.36748266	1.222919186	3.338150901	2.43E-36	5.95E-35
VCAN	1.00749474	1.001995871	1.013023787	0.007494917	PLA2G4D	0.115613662	0.005294935	4.448555212	7.56E-21	2.20E-20
GALK1	1.036378664	1.013594104	1.059675397	0.001630213	HS3ST5	0.023705017	0.222191209	-3.228537385	3.21E-19	8.40E-19
TREH	0.863363146	0.754959895	0.987331813	0.031856805	LDHC	0.208934469	0.570020052	-1.447962146	1.40E-13	2.69E-13
SLC25A19	1.364108558	1.166316309	1.595443828	0.000102377	AGXT2	14.88963982	32.33866855	-1.118951426	0.005501978	0.006422339

HIBADH	0.982212736	0.972927298	0.991586793	0.000212799
RPL21	0.993252228	0.988549976	0.997976847	0.00516731
XYLT2	1.054780597	1.007434385	1.104351931	0.02284211
SPTLC1	0.926172734	0.898694785	0.954490832	6.00E-07
CACNB3	1.269823621	1.182022686	1.364146433	6.39E-11
IL4I1	1.03439414	1.016399499	1.052707363	0.000158953
HAS3	1.339195201	1.124128545	1.595408101	0.001075566
MTTP	1.063482728	1.018555872	1.110391235	0.005192718
SLC44A1	0.864655105	0.81366757	0.918837714	2.74E-06
ENO1	0.999312601	0.998754091	0.999871423	0.015918921
APOM	0.989490212	0.9793591	0.999726126	0.044206342
MED26	2.318603122	1.436795836	3.741603577	0.00057257
UCK2	1.212531819	1.119729894	1.313025061	2.10E-06
NDST1	0.973329056	0.953718135	0.993343227	0.009238185
PNPLA8	0.879700559	0.816084661	0.948275479	0.000817761
AKR1B15	1.283855872	1.137321683	1.449269741	5.32E-05
BTD	0.768525768	0.69788082	0.846321949	8.72E-08
NUP62	1.123445858	1.055635166	1.195612496	0.000247874
FABP12	15352.13529	214.3218991	1099691.907	9.74E-06
CTSA	1.011810566	1.00442377	1.019251686	0.001685729
PIK3CA	0.814838337	0.731206338	0.908035779	0.000210594
PYGB	1.009707903	1.004770591	1.014669476	0.000112042
SEC24B	0.87758984	0.837624528	0.919462004	4.00E-08
PSMD11	1.114592839	1.059033656	1.173066777	3.20E-05
HAS2	1.23402023	1.138370303	1.337707005	3.25E-07
L2HGDH	0.695443722	0.587131866	0.823736537	2.61E-05
ENOPH1	0.963096273	0.934060673	0.993034454	0.016062127
TXNRD1	1.021765772	1.009459081	1.034222498	0.000496333
P4HB	1.001713839	1.000624	1.002804864	0.002048378
SLC9A1	1.098956012	1.057957949	1.141542836	1.15E-06
MTMR4	1.06523285	1.029888073	1.101790626	0.000242018
SGMS1	0.774896755	0.71157371	0.843854927	4.54E-09
SMS	1.00855796	1.003543005	1.013597975	0.000806459
MAN2C1	1.045690382	1.013478863	1.078925683	0.005131465
DGUOK	1.045110703	1.026944299	1.063598467	8.15E-07
PSMD8	1.019588837	1.004760162	1.03463636	0.009451643
LTA4H	0.94466926	0.913599719	0.976795409	0.00085009
NCOA2	0.894869829	0.838539449	0.954984302	0.00081256

STAB2	0.121113974	0.04560852	1.408990061	7.36E-12	1.33E-11
HADH	15.4898121	52.33342792	-1.756413117	6.35E-40	9.19E-38
RPL36A	17.41447553	7.488435861	1.21755071	1.26E-33	1.51E-32
GPD1L	6.675052335	18.81618064	-1.495122762	1.23E-35	2.39E-34
FABP5	5.110482824	1.390030232	1.878343339	3.53E-28	1.78E-27
RPL13	162.5812871	76.12893514	1.094644411	5.45E-29	2.98E-28
ACSBG2	0.093166002	0.302074302	-1.697027962	1.48E-08	2.29E-08
PLA2G4A	2.156617862	6.517388921	-1.595523526	8.22E-26	3.46E-25
LPL	7.88252081	18.19948001	-1.207168252	2.99E-09	4.80E-09
ACAN	4.431471681	0.723247813	2.615223933	2.78E-24	1.01E-23
HAO2	12.40163215	64.62680813	-2.381602737	3.14E-10	5.27E-10
ALDH4A1	16.63498608	68.44591693	-2.040743823	5.93E-19	1.52E-18
PCCA	7.331791955	19.67942326	-1.424450187	1.57E-32	1.50E-31
ACSF2	2.721778388	25.56426811	-3.231507213	4.86E-30	3.09E-29
RAPGEF4	3.679776594	1.796226444	1.034648943	2.66E-13	5.08E-13
LPIN3	9.995920641	3.327830181	1.586757633	1.88E-23	6.43E-23
CYP24A1	2.884707878	7.117859816	-1.303018289	4.15E-17	9.46E-17
ASS1	46.38766543	292.4275089	-2.656265887	2.32E-15	4.86E-15
BDH1	0.941491531	3.506545168	-1.897030289	5.52E-34	7.20E-33
PSAT1	7.969221281	44.95856672	-2.496085381	4.15E-24	1.48E-23
ALOX15B	1.511289479	0.078100228	4.274309451	2.52E-34	3.58E-33
PON3	0.30437273	0.716357046	-1.234839726	1.38E-21	4.17E-21
RPL37	75.98261772	37.75943	1.008832429	1.36E-31	1.06E-30
LPIN2	10.24070129	20.93879567	-1.03186395	4.63E-30	2.97E-29
ENO2	47.76765384	5.282017392	3.176873062	8.85E-37	2.87E-35
DPEP2	1.798371289	0.361325213	2.315321076	5.88E-37	2.08E-35
CYP2C8	1.538445777	0.410565796	1.90578825	6.34E-11	1.09E-10
PLIN2	208.070087	26.37278014	2.979948003	2.63E-31	1.96E-30
CHIT1	2.431162175	0.077507872	4.971159477	1.23E-21	3.72E-21
PDXK	10.24707435	21.94630433	-1.098765952	1.12E-21	3.41E-21
SLC6A12	10.77733745	24.70419853	-1.196755448	4.05E-06	5.61E-06
LIPI	0.113917819	0.01989316	2.517649077	0.000124788	0.000158958

SIN3A	0.83166474	0.749444981	0.922904626	0.000519394
CSAD	1.066367157	1.039150301	1.094296861	1.11E-06
UMPS	0.799730509	0.677033821	0.944663128	0.008541841
CHST11	1.071933661	1.042966914	1.101704912	6.70E-07
BHMT	0.993936798	0.990715224	0.997168849	0.000241033
PLCB3	1.095057019	1.036304354	1.157140631	0.001249161
VAPA	0.911828641	0.860166748	0.966593365	0.001923932
ADPGK	1.130413935	1.078304601	1.185041464	3.56E-07
PYCR1	1.053139762	1.03684747	1.069688059	7.58E-11
SMPD4	1.217998983	1.12066252	1.323789719	3.47E-06
ARSF	0.893097741	0.817461688	0.975732058	0.01227662
GNPDA2	0.750748532	0.61876513	0.910884165	0.00365895
RPL14	1.007103505	1.000551315	1.013698602	0.033547867
GCK	1.418679144	1.206107422	1.668715801	2.41E-05
PLD1	0.829700453	0.7343225	0.937466632	0.002732093
NADSYN1	1.151712607	1.073410115	1.235727063	8.43E-05
PARP4	0.959483528	0.93668322	0.982838831	0.000749885
HMGCS1	0.910131717	0.867571991	0.95477926	0.000116297
DBT	0.633743174	0.532174079	0.754697431	3.09E-07
BCO2	1.142691929	1.027903283	1.270299323	0.013530946
COASY	1.052752452	1.019050968	1.087568493	0.00195632
VAMP2	0.980237763	0.965424847	0.99527796	0.010193022
ARSI	1.085158802	1.038965285	1.133406132	0.000231206
SPHK1	1.082722451	1.050610998	1.115815376	2.29E-07
PLIN3	1.022935367	1.012065188	1.033922298	3.18E-05
PHKG1	1.09652405	1.000350792	1.20194336	0.049131049
ELOVL6	1.099091864	1.018110553	1.186514492	0.015537673
LMBRD1	0.974857736	0.965030304	0.984785246	8.40E-07
ADA	1.018993537	1.004234347	1.033969641	0.011484881
AKAP5	1.317744154	1.035612544	1.676736792	0.024791601
PI4K2A	1.065288779	1.009660301	1.123982176	0.020816158
ATIC	1.024113918	1.005769363	1.042793065	0.009772747
ASAH1	0.987793372	0.981439914	0.994187961	0.000191123
UQCRC2	0.983974892	0.971341271	0.996772831	0.014276819
G6PD	1.042294683	1.028228257	1.056553541	2.30E-09
UBIAD1	0.80904255	0.69689383	0.939238976	0.005380449
GLIPR1	1.021787394	1.000869723	1.043142234	0.041117758
PSME4	1.129569891	1.040355305	1.226434982	0.003702854

CYP21A2	1.236193293	1.128782136	1.353825338	4.83E-06
GAPDH	1.000326705	1.000115779	1.000537675	0.002397567
ARSA	1.027969582	1.007091434	1.049280558	0.008415266
ACADL	0.964314303	0.940811061	0.988404701	0.00389707
SLC25A2	9.47931187	3.00640123	29.88867641	0.000123715
ASRGL1	0.931245665	0.900782786	0.962738745	2.70E-05
SARDH	1.058049981	1.007492606	1.111144394	0.023897583
MARS	1.139764883	1.105188203	1.175423322	8.56E-17
SGSH	1.198289271	1.114918332	1.28789449	8.81E-07
CNDP2	0.990259592	0.986360702	0.994173895	1.16E-06
ELOVL7	0.963991955	0.947152846	0.98113044	4.53E-05
DAB1	1.159416082	1.083599384	1.240537482	1.81E-05
DSEL	0.907169763	0.826473399	0.995745271	0.040396931
PGAM1	0.980128111	0.967493797	0.992927413	0.002427917
ACER2	0.713099302	0.617497554	0.823502232	4.14E-06
FIG4	0.896442442	0.816156683	0.984625953	0.02239469
TYMP	1.014730475	1.009024129	1.020469092	3.73E-07
INPP5E	1.128294302	1.073368938	1.186030251	2.13E-06
SLC35B2	1.019000264	1.001742598	1.03655524	0.030792363
IYD	0.196203824	0.085859113	0.448361729	0.000112315
GNB3	1.34452291	1.171447866	1.54316885	2.55E-05
GATM	0.992191758	0.987430512	0.996975962	0.001403219
PSMD2	1.023768474	1.00971492	1.038017631	0.000865789
IDUA	1.157364037	1.107352296	1.209634476	8.91E-11
PIK3R3	0.927759055	0.898473535	0.957999128	4.61E-06
RPS15	1.002036364	1.000231771	1.003844213	0.026970414
ARG2	1.006427316	1.000893289	1.01199194	0.022765201
PLD6	1.158820412	1.009753322	1.329893865	0.035894589
AGPS	0.91951512	0.881520794	0.959147036	9.73E-05
NUDT15	0.918644833	0.86642435	0.974012711	0.004486345
PLA2G12A	0.927494415	0.879763374	0.977815077	0.005234782
GNAQ	0.934626848	0.907202761	0.962879945	8.61E-06
B3GAT3	1.051234088	1.034914289	1.067811237	3.87E-10
NUP93	1.385362093	1.183870109	1.621147551	4.81E-05
B3GAT1	1.690237005	1.359774061	2.101011642	2.26E-06
UQCRFS1	0.975095904	0.952198592	0.998543821	0.037510803
RPL22	0.985034247	0.975531033	0.994630037	0.002299435
GNA14	0.790498408	0.703672792	0.888037367	7.49E-05

OSBPL5	1.12485847	1.032393386	1.225605078	0.007179571
PPT1	0.988319266	0.980325943	0.996377764	0.004571177
IDI1	0.885018424	0.834865152	0.938184578	4.07E-05
ACACA	1.165561148	1.06631588	1.274043476	0.000740567
MED7	0.801659929	0.713493041	0.900721667	0.000200126
PLCB2	1.106045116	1.051796971	1.163091198	8.56E-05
SAT1	1.003615591	1.001633014	1.005602093	0.000347205
ACBD5	0.918734218	0.88448939	0.954304905	1.22E-05
WWTR1	0.980592951	0.968185685	0.993159216	0.002556909
AK1	1.206522008	1.095002671	1.329398908	0.000148203
ACHE	1.07960637	1.044196498	1.116217032	6.74E-06
RPL23	1.00400096	1.000334131	1.007681231	0.032442306
SLC5A1	0.975001505	0.961513001	0.988679232	0.000368343
CD38	1.089595825	1.021044896	1.162749128	0.009649517
RXRA	0.944627815	0.901134123	0.990220752	0.017856091
ETFA	0.951113596	0.922219974	0.98091247	0.001450706
MUT	0.927640105	0.900182898	0.955934806	9.60E-07
LMF2	1.019931867	1.005123697	1.034958201	0.008172682
SLC25A4	0.925436848	0.89075485	0.961469207	7.00E-05
EP300	0.920602246	0.886900492	0.955584649	1.38E-05
DBI	1.011624037	1.004270413	1.019031506	0.001904357
SRM	1.023907176	1.015672298	1.032208822	9.79E-09
NNT	0.965041321	0.939674807	0.991092603	0.008836651
CEPT1	0.830714299	0.732994711	0.941461427	0.003676292
PCK2	0.963416131	0.935588207	0.992071762	0.012694056
FPGS	1.053057815	1.018415699	1.088878306	0.00245205
DPEP1	0.950506758	0.91756154	0.984634881	0.004797897
ITPA	1.049518873	1.027658088	1.07184469	6.79E-06
CLTC	0.981733014	0.968837494	0.994800176	0.00628095
PPARGC1A	0.94544282	0.895322976	0.998368354	0.043515939
ST3GAL3	1.064384055	1.000697678	1.132123559	0.047465909
ALOX15	3.724418552	1.33052094	10.42546053	0.01228959
CHAT	1.186005044	1.074848277	1.308657227	0.00068005
PIP5K1A	1.070635284	1.018565454	1.125366962	0.007294058
VKORC1	1.013637284	1.007090964	1.020226157	4.18E-05
CCNC	0.92922311	0.877199791	0.984331731	0.012517833
ACMSD	0.977178074	0.965167449	0.98933816	0.000253478
AMD1	0.943064788	0.922565918	0.964019131	1.71E-07

KDSR	0.892042194	0.850228021	0.935912787	3.10E-06
TEAD4	1.08218374	1.051814552	1.113429783	5.38E-08
UPB1	0.925357871	0.881000228	0.971948886	0.001966911
SPTLC3	0.937519011	0.889997548	0.987577884	0.015059709
NUBPL	0.556240059	0.419388086	0.737748671	4.68E-05
PTDSS2	1.103576959	1.040986318	1.169930943	0.000938504
KHK	0.991394755	0.986403598	0.996411166	0.000790462
ESYT2	0.972484843	0.956433589	0.988805475	0.001017251
TRAP1	0.965069303	0.936289694	0.994733537	0.021345941
PDZD11	1.018197871	1.004675027	1.031902732	0.008200735
MTMR6	0.939270747	0.890214749	0.991030015	0.022068161
PEMT	1.012900495	1.003618581	1.022268253	0.006353121
MED1	0.9003064	0.836819275	0.968610114	0.004881232
AGL	0.796104561	0.719134436	0.881312924	1.11E-05
ARSD	0.944547685	0.915178934	0.974858899	0.000400236
RPL19	1.001035798	1.000075561	1.001996957	0.034491998
PANK3	0.889773428	0.829701361	0.954194834	0.001057945
ADIPOQ	1.143275259	1.052530395	1.241843774	0.00150706
ARL2BP	0.868681508	0.809156498	0.932585431	0.000101458
HPGD	0.923618316	0.858722176	0.993418847	0.032549161
CAD	1.187167689	1.112591499	1.266742665	2.18E-07
SDHC	0.91714104	0.865762891	0.971568192	0.003275957
PLEKHA3	0.70351753	0.496821994	0.996205727	0.047548358
B3GNT3	0.968161268	0.937409044	0.999922336	0.0494513
GNA15	1.153132486	1.098968386	1.209966135	6.45E-09
RGL1	0.953124727	0.935391733	0.971193899	5.43E-07
PLIN1	1.108763342	1.059445236	1.160377248	8.69E-06
RAB4A	0.906634363	0.860524346	0.955215123	0.000232854
ACAT1	0.975583189	0.964350951	0.986946253	2.87E-05
RPL36AL	0.997701037	0.995473628	0.99993343	0.043555518
GRHPR	0.93098452	0.90010859	0.96291957	3.24E-05
SAR1B	0.900972121	0.837303917	0.969481625	0.005289576
PHKB	0.833777045	0.766594053	0.906847839	2.22E-05
PYCR2	1.058998873	1.027130384	1.091856136	0.000235939
CALM1	0.978545416	0.969733815	0.987437086	2.61E-06
SLC35D2	0.945040059	0.912353777	0.978897371	0.001646351
ADCY2	0.546344714	0.351142112	0.850061944	0.007357742
PPP2CB	0.950881111	0.931303369	0.970870415	2.08E-06

PSMA5	1.074579472	1.027380068	1.12394729	0.001697429
LRPPRC	0.934816086	0.909655856	0.960672225	1.28E-06
CPT1B	1.430450659	1.212303854	1.687851673	2.23E-05
DLAT	0.906496748	0.865605247	0.949319977	3.07E-05
OSBPL3	1.04513882	1.001456992	1.090725974	0.042682652
ARNT	0.924440665	0.875509972	0.976106008	0.004632017
AASDHPPT	0.927283474	0.868466579	0.990083744	0.023943616
PSMB4	1.010438138	1.005361105	1.015540811	5.34E-05
SLC25A11	0.977535402	0.959834606	0.995562628	0.014811217
ANGPTL3	0.960842691	0.93086978	0.991780693	0.013496524
PSMC1	0.761892582	0.635488506	0.913439504	0.003301936
LUM	1.002918247	1.001499671	1.004338833	5.46E-05
CDK19	0.902077036	0.859014269	0.947298558	3.64E-05
GNG5	1.017574758	1.006804332	1.028460402	0.00133184
SLC27A3	1.055136088	1.014511774	1.097387129	0.007380213
NME4	1.021724464	1.013392685	1.030124744	2.68E-07
AMN	0.975315078	0.958641044	0.992279131	0.004498153
DARS	0.986148825	0.977482436	0.99489205	0.001954535
GDPD3	1.064966904	1.032422679	1.098536994	7.04E-05
LRP12	1.230715895	1.027121542	1.474666389	0.024448846
NMRAL1	1.031938564	1.004174556	1.060470208	0.023862274
AHR	0.977299054	0.957015077	0.998012951	0.031885979
CROT	0.780468564	0.671657148	0.906907909	0.001214315
NUP85	1.170402816	1.097713017	1.247906085	1.51E-06
DLST	0.977785762	0.959779899	0.996129422	0.017840606
CHDH	0.906159517	0.868940231	0.944973016	4.13E-06
HS6ST2	1.257320032	1.071261346	1.475693741	0.005071894
GNAS	1.008041786	1.004818533	1.011275378	9.50E-07
NQO1	1.01499479	1.006613443	1.023445922	0.000434714
DHTKD1	0.95489869	0.930372671	0.98007125	0.000508457
PCSK5	0.726304892	0.620853958	0.849666477	6.46E-05
HS2ST1	0.936302882	0.883635164	0.992109779	0.025871298
NPC1	1.049303839	1.020628811	1.078784503	0.00066328
MTM1	0.798157324	0.730010086	0.872666181	7.38E-07
ALDH9A1	0.980020361	0.968012866	0.9921768	0.001333703
CSNK2B	1.011319585	1.001234362	1.021506394	0.027721519
NMNAT3	0.801359243	0.688272845	0.933026256	0.004329403
PSMB2	1.068535447	1.034777607	1.103394579	5.18E-05

GALC	0.919859419	0.878983159	0.96263659	0.000315908
PSMD13	1.063500224	1.04304298	1.084358696	5.22E-10
NDST2	1.624676697	1.06768735	2.472235312	0.023467492
DDO	0.861607359	0.799525433	0.928509853	9.46E-05
LIPA	0.98934874	0.982279243	0.996469118	0.003425893
LRP1	1.014813811	1.001010539	1.02880742	0.035333701
NUP214	0.862602988	0.781587047	0.952016692	0.003312566
PSMB10	1.017570035	1.004758976	1.03054444	0.00705129
MTMR7	1.685427615	1.002248224	2.834294117	0.049018344
ACAD9	0.93661222	0.882865259	0.993631182	0.02986596
ACAA2	0.982249794	0.975585703	0.988959406	2.52E-07
ARSK	0.823674192	0.718445236	0.944315783	0.005410426
CHKB	1.231039942	1.13717454	1.332653243	2.80E-07
NUP153	0.943606371	0.901547176	0.987627721	0.012592142
PIK3R6	1.057895027	1.026716056	1.090020832	0.000226622
FAAH	0.964531335	0.939549048	0.990177892	0.006993023
GCG	2.348168835	1.024733067	5.380812871	0.043621103
DIO2	1.055571246	1.015241571	1.097502985	0.006507958
ACOT9	1.195749818	1.092263191	1.3090413	0.000108486
AP2B1	0.970607018	0.954273279	0.987220332	0.00057035
RPS3A	0.997646259	0.995311121	0.999986875	0.048730601
PRKD1	0.826537413	0.759647965	0.899316693	9.66E-06
DCK	0.947136108	0.899336098	0.997476704	0.039822554
RRM2	1.091652673	1.062729927	1.121362566	1.54E-10
PLA2G2A	1.038546453	1.019941895	1.057490373	4.12E-05
GNB5	0.641481863	0.515233071	0.798665699	7.17E-05
PSMB6	1.007062666	1.001321107	1.012837148	0.015842094
SEH1L	0.869405838	0.759471707	0.995253023	0.042463348
PPARA	0.834168204	0.760215539	0.915314879	0.000129098
PEX11A	0.896258514	0.836073051	0.960776482	0.002013921
ACBD7	1.675358161	1.218944811	2.302667803	0.001472389
PIK3CD	1.065768938	1.012458315	1.121886612	0.014979894
PHYH	0.962770867	0.940627723	0.985435278	0.001394267
SLC22A13	0.80186452	0.663588036	0.968954643	0.022223114
UCP2	1.008207322	1.00200686	1.014446151	0.009406088
CHST9	0.881623173	0.805456549	0.964992364	0.006276989
GLO1	0.987952602	0.976312777	0.9997312	0.045024834
ENPP3	0.994628019	0.991284479	0.997982837	0.001716964

GLUD2	0.548872899	0.447025769	0.673924146	1.01E-08
TEAD1	0.922775575	0.883718657	0.963558656	0.000270186
DLD	0.964294582	0.941229556	0.987924823	0.003245333
ACAA1	0.926848371	0.867581836	0.99016354	0.024248948
NCOA3	0.918686242	0.869690551	0.970442198	0.002422005
AMY2B	1.18296279	1.085579941	1.289081448	0.000126393
CHST2	1.030429767	1.00466138	1.056859082	0.020347611
PKLR	0.976562895	0.956219317	0.997339283	0.027243478
THTPA	0.699857826	0.601793006	0.813902739	3.60E-06
NUDT16	0.949515916	0.907380831	0.993607584	0.025294345
ALDH1A1	0.99812624	0.996607998	0.999646794	0.015743314
DCTPP1	0.956074628	0.929078691	0.983854978	0.002113807
GLUD1	0.990660148	0.984623423	0.996733884	0.002621171
B3GNT4	1.081054886	1.000458768	1.16814376	0.048659093
LARS	0.962262314	0.927217188	0.998632007	0.042124824
GPC4	0.975995317	0.956938298	0.995431849	0.01573274
FDX1	0.878457938	0.819645265	0.941490644	0.000247128
LPA	0.005578105	0.000143627	0.216638618	0.00544968
INPP4B	0.848243662	0.765741281	0.939635002	0.001618235
RPIA	1.096127928	1.032788757	1.163351582	0.002508321
CYP1B1	1.004119897	1.000461907	1.007791262	0.027246724
FADS2	1.036682776	1.003341159	1.071132355	0.030776504
CHD9	0.815139869	0.743074281	0.894194596	1.51E-05
SLC25A20	0.927142469	0.873829682	0.983707897	0.01229379
NME2	1.022842255	1.011593047	1.034216558	6.26E-05
GNAI1	0.961289664	0.927443391	0.99637113	0.03086935
ACADM	0.936737806	0.917702604	0.956167841	4.40E-10
RPL29	1.001402173	1.000261762	1.002543884	0.015945677
SERINC3	0.978392367	0.965215866	0.991748746	0.00159054
TIAM2	1.61217444	1.045627118	2.485691485	0.030623086
CYB5A	0.984788972	0.975721376	0.993940836	0.001163456
DDHD1	1.534856652	1.145899177	2.05583963	0.004061785
RAN	1.020458468	1.008350564	1.03271176	0.000882693
PLA2G4D	1.544487896	1.130911175	2.109310541	0.006264379
COL4A3BP	0.866275422	0.814731508	0.921080258	4.51E-06
AGXT2	0.971114405	0.957599398	0.984820154	4.15E-05
GCLC	0.85838136	0.784566547	0.939140934	0.000872773
HADH	0.923966911	0.893089515	0.955911853	5.12E-06

RPL36A	1.010890879	1.003119456	1.01872251	0.005941919
UCP3	1.59207227	1.177165345	2.153218427	0.002537864
MTHFS	0.85846926	0.789906316	0.932983388	0.000326436
MED28	0.862519976	0.745837759	0.997456485	0.046118677
FABP5	1.04817923	1.026180329	1.070649735	1.37E-05
RPL13	1.001464937	1.000421748	1.002509214	0.005906403
BPGM	0.959678545	0.927823949	0.992626792	0.016864273
B4GALT2	1.056375036	1.028153323	1.085371405	7.20E-05
CSPG5	3.462532193	1.84044169	6.514267337	0.000117284
CYP4F11	1.040909888	1.010288097	1.072459825	0.008492996
SLC5A10	0.962428263	0.940888838	0.984460782	0.000912843
ZDHHC8	1.083309282	1.04788473	1.119931389	2.39E-06
ACBD4	0.95627679	0.917254673	0.996958996	0.035444505
PSMD4	1.027281439	1.013778446	1.040964285	6.69E-05
MED13L	0.938041296	0.89395285	0.984304119	0.009212686
LPL	0.966925258	0.941080978	0.993479283	0.014964038
SPTLC2	0.945916631	0.912272386	0.980801663	0.002620559
GNG3	5.169663207	2.695051585	9.916477228	7.69E-07
SH3KBP1	1.076908193	1.034869584	1.1206545	0.000265244
HAO2	0.969271513	0.955174559	0.983576518	2.98E-05
PCCA	0.871205153	0.828110504	0.916542437	9.99E-08
RAPGEF4	0.822582736	0.756977746	0.893873514	4.11E-06
LPIN3	1.031625196	1.014769011	1.048761376	0.000212069
NDUFA5	0.939336596	0.894202534	0.986748759	0.012740947
NUP37	1.188795244	1.057750823	1.336074718	0.003706255
PPP2R1B	0.888404285	0.810916586	0.973296374	0.011045522
PSMB1	1.00782852	1.000244734	1.015469806	0.043025685
PSMD7	1.067916965	1.026970166	1.110496372	0.000987425
ACSL1	0.98966095	0.982992981	0.99637415	0.002586192
PSAT1	1.016371038	1.009875473	1.022908382	6.90E-07
MED25	1.149957688	1.088382267	1.21501675	6.48E-07
GCKR	1.188387744	1.043229975	1.353743148	0.009412927
RPL37	1.005698275	1.002225687	1.009182895	0.001283074
PHKG2	1.306698187	1.198761063	1.42435403	1.19E-09
SLCO1B3	4.7790847	2.450070269	9.322038988	4.46E-06
ENO2	1.008150681	1.003965725	1.012353082	0.000130874
MYLIP	0.940860902	0.920220938	0.961963807	7.19E-08
GPIHBP1	0.961057946	0.926779121	0.996604644	0.03207259

PLIN2	0.998261713	0.997178734	0.999345869	0.001680901
INSIG2	0.978697652	0.95937014	0.998414537	0.034354814
LRP8	2.46216783	1.964514772	3.085886912	5.23E-15
MED21	0.883688152	0.828896334	0.942101827	0.000152966
ALDH7A1	0.930037	0.899173263	0.961960121	2.53E-05
OSBPL1A	0.881275729	0.846421985	0.917564672	8.32E-10
